# The Structure of Social Networks and Its Link to Higher Education Students’ Socio-Emotional Loneliness During COVID-19

**DOI:** 10.3389/fpsyg.2021.733867

**Published:** 2022-01-13

**Authors:** Manuel D. S. Hopp, Marion Händel, Svenja Bedenlier, Michaela Glaeser-Zikuda, Rudolf Kammerl, Bärbel Kopp, Albert Ziegler

**Affiliations:** ^1^Department of Psychology, University of Erlangen Nuremberg, Erlangen, Germany; ^2^Department of Education, University of Erlangen Nuremberg, Erlangen, Germany

**Keywords:** COVID-19 pandemic, loneliness, social network, interconnectedness, network density, network homogeneity, network heterogeneity, digital information-sharing behavior

## Abstract

Lonely students typically underperform academically. According to several studies, the COVID-19 pandemic is an important risk factor for increases in loneliness, as the contact restrictions and the switch to mainly online classes potentially burden the students. The previously familiar academic environment (campus), as well as the exchange with peers and lecturers on site, were no longer made available. In our cross-sectional study, we examine factors that could potentially counteract the development of higher education student loneliness during the COVID-19 pandemic from a social network perspective. During the semester, *N* = 283 students from across all institutional faculties of a German comprehensive university took part in an online survey. We surveyed their social and emotional experiences of loneliness, their self-reported digital information-sharing behavior, and their current egocentric networks. Here, we distinguished between close online contacts (i.e., mainly online exchanges) and close offline contacts (i.e., mainly in-person face-to-face exchanges). In addition, we derived the interconnectedness (i.e., the densities of the egocentric networks) and heterogeneity (operationalized with the entropy) of students’ contacts. To obtain the latter, we used a novel two-step method combining t-distributed stochastic neighbor embedding (t-SNE) and cluster analysis. We explored the associations of the aforementioned predictors (i.e., information-sharing behavior, number of online and offline contacts, as well as interconnectedness and heterogeneity of the close contacts network) on social and emotional loneliness separately using two hierarchical multiple linear regression models. Our results suggest that social loneliness is strongly related to digital information-sharing behavior and the network structure of close contacts. In particular, high information-sharing behavior, high number of close contacts (whether offline or online), a highly interconnected network, and a homogeneous structure of close contacts were associated with low social loneliness. Emotional loneliness, on the other hand, was mainly related to network homogeneity, in the sense that students with homogeneous close contacts networks experienced low emotional loneliness. Overall, our study highlights the central role of students’ close social network on feelings of loneliness in the context of COVID-19 restrictions. Limitations and implications are discussed.

## Introduction

In early 2020, the new COVID-19 brought drastic transformations to the lives of many people around the world. Many nations enacted far-reaching COVID-19 measures, such as masking mandates and restricting in-person face-to-face contacts. The resulting school and university closures affected over 1.5 billion students and adolescents, according to UNESCO (2021). Higher education institutions across many countries switched to online distance education at short notice. This so-called emergency remote teaching (Hodges et al., 2020) had a profound impact on teaching. The learning spaces transformed from shared seminar rooms and library space to the personal confines of the home. In-person teaching was replaced by asynchronous and synchronous online courses. This change from offline teaching to online teaching posed new challenges for the digital skills of the students. Online tools had to be operated; reliable webcam and microphone communication had to be established; and online information exchange with other peers had to be ensured. Students who could not meet these requirements could fail to catch up. Not only was teaching restricted, so, too, were regular in-person meetings with other students and friends. The COVID-19 restrictions greatly limited the opportunities to meet others in-person not only on campus and in seminars, but also in the context of students’ private lives. In many countries, contact with more than one person outside the household was prohibited. This led to an increased feeling of loneliness (Liu et al., 2021)—especially when the exchange with close contacts (i.e., the people with whom they discuss sensitive topics) was no longer possible in-person (Russell et al., 2012).

The aim of the study is to explore possible factors influencing students’ feelings of loneliness in a German sample. We hypothesize that an aspect of digital skills, i.e., the ability to easily exchange information with peers called “information-sharing behavior,” have a lowering effect on the perception of loneliness. Furthermore, we focus on the close social network of the students (i.e., the people with whom they discuss sensitive topics) and investigate the connections between their structures and students’ perceptions of loneliness. For the first network-related question, we examine whether the way in which students communicate with their close contacts has a varying impact on their sense of loneliness. Here we distinguish between the number of contacts with whom communication took place mainly online and the number of contacts with whom communication took place mainly offline, i.e., in-person. The second network-related question concerns the connectivity structure of the network, namely, whether greater interconnectivity in the close social network is helpful to students’ sense of loneliness. The last question addresses the diversity of the network, i.e., whether it is more helpful to have many close contacts of a similar type or whether a high degree of diversity is more helpful. Here, we implicitly consider the mechanisms of social network formation (i.e., selection and influence effects).

## Theoretical Background

### COVID-19 Pandemic Restrictions and the Feeling of Loneliness

Worldwide the COVID-19 pandemic had a far-reaching impact on higher education teaching. In 165 countries, schools and universities were closed (UNESCO, 2020), and a sudden switch from mainly offline teaching to a special form of online teaching occurred. Due to its differences from conventional online teaching, the term *emergency remote teaching* was coined (Hodges et al., 2020). For the more than 1.5 billion affected students and adolescents, this meant a blatant rift in their social environment. Alongside the extracurricular contact restrictions, students lost many opportunities to meet and interact with peers and other close contacts outside of their own households. For example, in many countries, contact with more than one person outside the household was prohibited. As a result, many contacts were eliminated, and increasing feelings of loneliness had to be confronted (Bu et al., 2020; Liu et al., 2021).

Loneliness has harmful effects on academic achievement. In particular, evidence suggests that feelings of loneliness can have a negative impact on grades (Neto et al., 2015; Rosenstreich and Margalit, 2015) and are associated with an increased attrition rate (Rotenberg and Morrison, 1993; Alkan, 2014). Loneliness can affect academic grades through multiple impact pathways. For one, studies show that loneliness leads to a decrease in self-efficacy (Fry and Debats, 2002; Al Khatib, 2012), which itself affects the academic performance (Honicke and Broadbent, 2016). Loneliness can also lead to so-called learning burnout (Lin and Huang, 2012), where the students experience emotional exhaustion and negative attitudes toward their learning and university activities (Schaufeli et al., 2002).

The increased dropout rate of lone students (i.e., student attrition), is described by Tinto’s (1993) student integration model. His concept implies that student attrition is associated with the student’s perceived (person-environment) fit to the university environment, i.e., feeling lonely represents a suboptimal fit. Rotenberg and Morrison (1993) repeatedly measured the loneliness of freshman students on two consecutive fall semesters and were able to show that loneliness predicted attrition, even after controlling for poor grade point average. This finding is supported by additional research indicating that positive social relationships make a substantial contribution to explaining students’ academic satisfaction, which is a key indicator of student attrition (Bernholt et al., 2018). Overall, literature suggests that there is a connection (albeit moderate; Rotenberg and Morrison, 1993) between experience of loneliness and academic performance.

### Loneliness as a Multidimensional Construct

Loneliness is understood as the subjectively perceived discrepancy between desired and actual social relationships (Weiss, 1973). According to Weiss’ topology, loneliness is a multidimensional construct. It is characterized by two aspects: social loneliness and emotional loneliness. *Social loneliness* refers to the number of relationships that is less than the desired number. For example, people who have recently moved (e.g., to a new city or university) are likely to experience this form of loneliness (Russell et al., 1984). *Emotional loneliness*, on the other hand, refers to situations in which the absence of closeness and intimate relationships is lamented. For example, people who have recently been widowed or had a romantic relationship broken off often experience this form of loneliness (Russell et al., 1984). This proposed two-dimensional nature of loneliness means that someone can feel lonely even though they have many friends (i.e., low social but high emotional loneliness; Weiss, 1973). The involuntary constraints of the COVID-19 pandemic led to a reduction of the (close) social network, which in turn had a direct impact on the emotional as well as the social sense of loneliness of both young and old adults (Killgore et al., 2020; Liu et al., 2021).

### Factors Related to Loneliness During COVID-19 Restrictions

#### Digital Information-Sharing Behavior and the Close Social Network

During the COVID-19 pandemic, the importance of digital skills was twofold. First, the newly introduced emergency remote teaching placed demands on students’ digital skills. Students were required to use online tools and to exchange information efficiently with fellow students and lecturers so as to avoid falling behind the lecture—what Hong and Kim (2018) call information-sharing behavior.

Second, the pandemic restrictions posed new challenges to communication with friends and fellow students, whose presence plays an important aspect in feelings of loneliness (Russell et al., 2012). Due to the loss of in-person meetings, it was now essential for higher education students to be able to efficiently exchange information with close contacts via common (digital) communication tools (i.e., telephone, video chat, messenger) to avoid losing touch. The ability to exchange information is therefore an essential building block for remote teaching, as well as for maintaining one’s online social network.

The widespread adoption of the internet in many people’s daily lives created new ways of communicating with their contacts (e.g., via chat, voice, or video chat). This raises the question of whether online communication can even serve as a substitute for in-person face-to-face contact and if, to which extent has it an impact on feelings of loneliness?

Some researchers argued that online communication could have a negative impact on people’s well-being because it displaces time that could be spent with friends in-person (e.g., Kraut et al., 1998). Other researchers reasoned that online communication could increase the quality of relationships with friends and therefore one’s own well-being (e.g., Valkenburg and Peter, 2007b). While there is evidence for both hypotheses (e.g., Kraut et al., 2002), it seems to be of great importance with whom, rather than whether, people communicate online (for a concise literature review, see Valkenburg and Peter, 2007a). If the exchange takes place, for example, with friends and other close contacts (i.e., “strong ties” according to Granovetter, 1973), positive effects on relationship quality as well as on feelings of loneliness were shown, especially with new communication methods, such as video chat (Shaw and Gant, 2002; Manago et al., 2020; Nakagomi et al., 2020). This association does not tend to occur in the case of online exchanges with casual acquaintances (i.e., “weak ties”; Valkenburg and Peter, 2007b).

The preceding findings are strengthened by further loneliness research, since a central decisive factor for the perception of loneliness is the immediate social network (i.e., a person’s close contacts) and the social support that it provides (Nicpon et al., 2006). Lonely individuals have a lower number of close contacts (Shin, 2007) and are less likely to interact with them than non-lonely individuals and thus, may experience lower levels of social support (Russell, 1982). Jackson et al. (2000) showed a direct link between social support and loneliness among college students. Low levels of social support during the semester predicted feelings of loneliness at the end of the semester. This also applies in the online context, where a lower perceived social support is associated with a lower number of online contacts (Nabi et al., 2013; Dai et al., 2021), all of which can affect the feeling of loneliness (Moody, 2001).

Overall, previous research indicates that, in the context of COVID-19 restrictions, individuals who are more capable of communicating seamlessly online with their existing close circle might be better protected from feelings of loneliness (Valkenburg and Peter, 2007b; Kralj Novak et al., 2015; Kluck et al., 2021). This effect is further enhanced by a higher number of close contacts, which could also translate to online exchange (Shin, 2007; Nabi et al., 2013; Dai et al., 2021).

#### Interconnectivity of Close Contacts

Along with the number of social relationships, the interconnectedness of the circle of contacts is an important factor in the individual’s sense of loneliness (Stokes and Levin, 1986; Bell, 1991; Kovacs et al., 2021). As described above, the circle of family and close friends might be particularly protective against feelings of loneliness. This close network, also sometimes referred to as bonding capital, is characterized by a relatively high degree of interconnectivity (Salehi et al., 2019). In network research, interconnectedness is expressed in terms of network density which indicates the ratio between existing links in the present network and the theoretical maximum number of links (i.e., everyone is connected to everyone else). An early cross-sectional study by Stokes (1985) supports that a high interconnectivity (i.e., high density) of an individual’s close contacts can be an important correlate of low feelings of loneliness. In Stoke’s case, interconnectivity turned out to be even more relevant than the number of close contacts. However, the favorable link between interconnectivity and feelings of loneliness does not appear to be unconditional. Other research suggests that loneliness may spread through social networks, much like a viral infectious disease (Cacioppo et al., 2009); therefore, the “contagiousness” of loneliness should be taken into account, particularly in longitudinal study designs. However, as shown by Stokes (1985), a loneliness-reducing effect of interconnectivity dominates in cross-sectional designs.

#### Diversity of Close Contacts

A further aspect that can contribute to an individual’s feeling of loneliness is the homogeneity or diversity of the network actors. Here we refer to homogeneous networks if the network actors are very similar to each other (e.g., in terms of behavior or attitudes), and to heterogeneous networks if they are very dissimilar to each other. Homogeneous networks are often a result of mainly two processes: selection and influence (Snijders, 2001).

Selection means that contacts outside the family circle are selected according to one’s own preferences, i.e., new contacts who have similar characteristics to oneself are favored. This process is based on homophily (McPherson et al., 2001), which can be expressed with the phrase “birds of a feather flock together.” Selection processes are observed in the offline school context (Burk et al., 2008; Steglich et al., 2010; Hopp et al., 2019), in university (Mayer and Puller, 2008; Smirnov and Thurner, 2017), as well as in online contexts (e.g., in online social networks; Mayer and Puller, 2008; as well as in online mentoring; Hopp et al., 2020). Choosing new contacts selectively can lead to homogeneous individual networks (Mayer and Puller, 2008; and clusters in the global network; Cacioppo et al., 2009; Hofstra et al., 2017). Additional to selection, influence processes occur between people in the same network and can increase homogeneity of the network. Here, behaviors (e.g., smoking) or attitudes (e.g., confidence) can spread between the actors in the network. Influence processes have been identified in many contexts—offline and online (e.g., Mercken et al., 2010; Caravita et al., 2014; Hopp et al., 2020). Moreover, there is evidence that loneliness can also spread through influence processes—especially through close friends (Cacioppo et al., 2009).

Research to date indicates a desirable role of homogeneous close contacts networks in terms of low feelings of loneliness, although studies so far have been rather limited. Homogeneous networks often consist of close contacts, such as family and close friends, whereas in heterogeneous networks, links between the actors tend to be rather weak (Coffé and Geys, 2007). Especially family and close friends play a major role in the feeling of loneliness (Weiss, 1973). Some studies on bonding capital (i.e., the network consisting of close contacts) underline a protective effect of homogeneity on feelings of loneliness (Simons et al., 2020; Thomas et al., 2020). Few studies have explicitly investigated the homogeneity of the network based on different types of actors. Van Baarsen et al. (1999) examined different types of relationships (e.g., parent, child, friend, etc.) and could demonstrate this protective association in a sample of Dutch elderly. Ashida and Heaney (2008) showed that in older adults, more homogeneous networks in terms of the network members’ demographic characteristics could improve social support and therefore people experience less feelings of loneliness (Russell, 1982). However, contradicting research in older subpopulations indicates that homogeneous network connections might increase the risk of loneliness, due to the reduced resilience that homogeneous networks might be associated with (Robustelli et al., 2017; Liebke, 2019).

Nevertheless, due to the unusual situation of COVID-19 constraints, homogeneous networks (indicating high social support) may be predominantly related to perceptions of low loneliness (evidence of limited comparability, Benkel et al., 2009).

### The Current Study

The COVID-19 pandemic and the resulting restrictions on face-to-face interactions present a novel research opportunity to explore associations between one’s social network and one’s sense of loneliness. We investigate to what extent digital information-sharing behavior and the structure of close contact networks help to mitigate feelings of loneliness in higher education students. Here, we examine a sample of German students who were exposed to the above-mentioned COVID-19 restrictions during the study period.

Digital information-sharing behavior should make it easier to deal with contact restrictions; that is, students demonstrate the ability to communicate and interact via online tools. Therefore, our first hypothesis is:

***Hypothesis 1***. In the given context of COVID-19 restrictions, digital information-sharing behavior is associated with lower feelings of loneliness, both in terms of social loneliness (H1a) and emotional loneliness (H1b).

The immediate social network (i.e., close contacts) is a central factor that plays a decisive role in the perception of loneliness (Nicpon et al., 2006). We consider three structural measures, i.e., number of close contacts, interconnectivity of the social network, and homogeneity of the social network members. We distinguish between offline and online contacts to examine how the number of online contacts affects students’ feelings of loneliness. Therefore, we investigate the following hypothesis:

***Hypothesis 2.*** In the given context of COVID-19 restrictions, a higher number of online contacts are associated with lower levels of loneliness, both in terms of social loneliness (H2a) and emotional loneliness (H2b).

Beyond the number of contacts, the interconnectivity of the contacts is also likely to contribute to a supportive network and could thus be associated with a low sense of loneliness.

***Hypothesis 3.*** In the given context of COVID-19 restrictions, higher interconnectivity of all close contacts is associated with lower social (H3a) and lower emotional (H3b) loneliness.

Finally, we argued that homogeneous networks are linked with low feelings of loneliness. Therefore, we investigate the fourth hypothesis:

***Hypothesis 4.*** In the given context of COVID-19 restrictions, higher homogeneity of contact types is associated with lower social (H4a) and lower emotional (H4b) loneliness.

## Materials and Methods

### Sample

In total, the raw data set consisted of 363 students enrolled at the University of Erlangen-Nuremberg, Germany. The performed data cleaning (see section “Plan of Analysis”) resulted in 283 listwise complete cases (78.0%). Comparison between the two samples (100 and 78.0%) led to no significant differences (*t*-tests, all | *t*| < 0.78, all *p* > 0.44) regarding all variables depicted in Table 1.

**TABLE 1:** Descriptives (*n* = 283).

	*M*	*SD*	Median	Mad	Min	Max	Skew	Kurtosis
Age	23.48	5.06	22.00	2.97	18.00	59.00	3.30	15.27
Gender*	1.72	0.46	2.00	0.00	1.00	2.00	–0.89	–0.90
Semester	4.54	2.74	4.00	2.97	1.00	15.00	0.99	0.85
Social loneliness	2.39	1.03	2.20	1.19	1.00	5.40	0.57	–0.37
Emotional loneliness	3.06	1.01	3.00	0.99	1.00	6.00	0.15	–0.34
Offline contacts	2.72	1.57	3.00	1.48	0.00	8.00	0.49	–0.07
Partner	0.54	0.50	1.00	0.00	0.00	1.00	–0.15	–1.99
Inf.-sharing beh.	5.29	0.85	5.50	0.74	1.75	6.00	–1.52	2.57
Online contacts	2.64	1.74	3.00	1.48	0.00	8.00	0.62	0.21
Interconnectedness	0.68	0.29	0.70	0.40	0.00	1.00	–0.58	–0.54
Heterogeneity	1.13	0.41	1.15	0.34	0.00	1.91	–0.80	0.55

Mad, median of absolute deviation. Partner refers to current significant other. Both interconnectedness and heterogeneity refer to all contacts, regardless of online or offline. *Gender coding: 1 = “male,” 2 = “female.” Inf.-sharing beh. stands for information-sharing behavior.

Participants were members of the following university faculties: 34.1% faculty of humanities, social sciences, and theology, 18.6% faculty of business, economics, and law, 17.6% faculty of engineering, 16.1% faculty of medicine, and 13.6% faculty of sciences. The participants were between 18 and 59 years old (*M*_age_ = 23.5 years), and were predominantly female (i.e., 72%). On average, students were in the middle of their fourth semester (*M*_semester_ = 4.54). For more details (see Table 1).

### Procedure

The data collection was conducted during the summer semester in 2020 at the University of Erlangen-Nuremberg in Germany as an online survey with one measurement. At that time, Germany was subject to the restrictions described in the introductory section, such as lockdown orders, and higher education teaching was mainly online. All enrolled students were notified about the questionnaire via email from an official university channel. They were informed that the online survey will take approximately 12 min and that it is about students’ personal social network and that the results might help to better understand the changes in student life due to (the COVID-19) contact restrictions. The questionnaire was implemented in the German language using the online survey system Unipark Questback EFS (unipark.com). After answering demographic questions and the batteries regarding loneliness and digital information-sharing behavior, participants were asked to name up to eight close contacts with whom they had “discussed matters important” to them in the last 4 weeks. Here, the participants were instructed not to use names that would allow conclusions to be drawn about the contacts named. Then the students had to answer for each given contact the following items. For distinguishing “offline” from “online” contacts, they had to provide the main channel of communication during the past 4 weeks. For deriving the students’ contacts’ heterogeneity, students provided the contacts’ initiation of exchange, gender, residence, relationship to student, social attraction, and media skill, see Supplementary Appendix for more details. To determine the interconnectivity between the subjects’ contacts, we presented participants with an upper triangular matrix (based on the previously mentioned close contacts) in which we indicated to them, step-by-step, that they could mark which contacts knew each other by checking boxes. After 2 weeks, students were reminded again to complete the questionnaire. Subsequently, the questionnaire data were extracted and subjected to further data processing. In accordance with the institutional commissioner for data protection, participants’ privacy was protected; all data has been anonymized; and participating students were not disadvantaged due to non-participation. Informed consent of the participants was obtained by virtue of survey completion.

### Measures

#### Social and Emotional Loneliness

To assess social and emotional loneliness, the Loneliness Scale developed by Jong Gierveld and colleagues was used (Jong Gierveld and Kamphuls, 1985; de Jong Gierveld and Van Tilburg, 1999). The total 11 item scale consists of separate social (5 items) and emotional (6 items) loneliness subscales, and is demonstrated to be valid and reliable measurement instruments for these phenomena (van Baarsen et al., 2001; Dykstra and Jong Gierveld, 2004). They were measured using a six-point Likert scale with 1 = “not at all true” to 6 = “completely true,” and were recoded, if necessary. A high scale value indicates high-perceived loneliness.

##### Social Loneliness

The social loneliness subscale (e.g., “there are enough people I feel close to,” recoded) showed good internal consistency indicated by Cronbach’s α = 0.88. The subscale showed a good one-dimensionality, indicated by McDonald’s ω_h_ = 0.84, which gives the proportion of variance in scale scores accounted for by a general factor (McDonald, 1999). A high ω total value of McDonald’s ω_t_ = 0.91 indicated a reliable multidimensional composite (Watkins, 2017).

##### Emotional Loneliness

The emotional loneliness subscale (e.g., “I miss having people around”) showed acceptable internal consistency indicated by Cronbach’s α = 0.78. The subscale showed an acceptable one-dimensionality, indicated by McDonald’s ω_h_ = 0.66 (McDonald, 1999). Again, a high ω total value of McDonald’s ω_t_ = 0.89 indicated a reliable multidimensional composite (Watkins, 2017).

#### Information-Sharing Behavior

We used the “Information-Sharing Behavior” subscale of the measurement tool “Readiness for Academic Engagement Scale” (Hong and Kim, 2018). The subscale consisted of four items (e.g., “I can interact with classmates using real-time communication tools, for example, video conferencing tools or messengers”) and used a six-point Likert scale with 1 = “not at all true” to 6 = “completely true.” The subscale showed good internal consistency indicated by Cronbach’s α = 0.83. The McDonald’s hierarchical ω indicated good one-dimensionality with ω_h_ = 0.79 (McDonald, 1999). A high McDonald’s total ω value of ω_t_ = 0.87 indicated a reliable multidimensional composite (Watkins, 2017).

#### The Online Exchange With the Social Network of Close Contacts

Participants were asked to name up to eight close contacts with whom they had “discussed matters important” to them, which is based on Marsden’s (1987) name generator. For each contact mentioned, they were also asked to indicate whether the exchange occurred predominately online (e.g., video chat, instant messenger) or predominately offline (i.e., in-person face-to-face). Thus, a student could have up to eight close contacts with varying numbers of online and offline contacts (e.g., two offline and six online contacts, or one offline and three online contacts).

#### Interconnectivity of Close Contacts

Interconnectivity describes the extent to which a student’s contacts know each other. The interconnectivity was operationalized with the network measure density, which represents the ratio of observed connections to the maximum possible connections. It is calculated with the formula (2 **×** *d*)/(*N* **×** (*N*–1)), where *N* is the number of all contacts in the network and *d* the observed connections between the contacts. The value ranges from 0 (i.e., no one knows each other) to 1 (i.e., all the contacts know each other). For example, if a student has three contacts, and two contacts know each other, then the interconnectivity is 1/3 (because one of three possible connections is realized).

#### Heterogeneity of Close Contacts

As the measure of network heterogeneity, we chose the Shannon entropy (also called Shannon index; Jost, 2006) of the close contact types of each student. The Shannon entropy is a widely used, reliable measurement of homogeneity or heterogeneity (Jost, 2006; Masisi et al., 2008)^1^. For calculating the entropy, we derived the types of close contacts using a two-step procedure. Here, we collected additional variables for each contact (e.g., closeness to person and residence) and applied a combination of t-distributed stochastic neighbor embedding (t-SNE; i.e., step 1) and cluster analysis (i.e., step 2) to derive 12 types. A detailed description of the used variables and process of analysis can be found in Supplementary Appendix.

For each participant, the Shannon entropy is defined as the negative of the sum of the probability of each close contact type multiplied by the logarithm of the probability of each close contact type. A high entropy value reflects high heterogeneity, and a low entropy value reflects low heterogeneity (i.e., high homogeneity). The numerical value of entropy is determined by two properties, by the number of types and their probability distribution. The entropy increases with the number of types and with an equal distribution of these types. If the number of types is given (e.g., the eight close contacts consist of two types “type A” and “type B”), the Shannon entropy reaches its maximum when all types are occupied with equal frequency (e.g., four contacts are “type A” and four contacts are “type B”)—regardless of the order of the types. We found twelve different types of close contacts; thus, entropy theoretically could take values between zero and log(12) = 2.5. However, since only a maximum of eight contacts could be named, the entropy was limited to log(8) = 2.1.

### Plan of Analysis

#### Data Preparation

The data were available as an SPSS file and were prepared for the following steps using SPSS v26 (IBM Corp, 2019): Definition of missing values, recoding of negatively worded items, and calculation of scales. Subsequently, further processing of the data took place in R v4.0.4 (R Core Team, 2020). The data set was examined for duplicates, and individuals who had more than 90% missing values in the dependent or independent variables were removed (*n* = 13, 3.6%). In addition, *n* = 67 (18.5%) cases showed missing values in the dependent or independent variables and were excluded from further analysis. Subsequently, we calculated the variables: offline and online contacts, interconnectedness, and heterogeneity, as reported in section “Measures.”

#### Data Analytic Strategy

We began our data analysis by examining the descriptive statistics and the bivariate Pearson correlation to provide a first impression of the structure of the variables of interest. This was followed by our main analysis consisting of hierarchical regressions with social and emotional loneliness as criteria. The control variables were age, gender, and the presence of a partner (derived from the variable “relationship to student” of the indicated close contacts). We built the hierarchical regression on a base model with the independent variables gender, age, offline contacts, partner, and the corresponding loneliness subscale as the dependent variable.

First, to test hypotheses H1a and H1b, i.e., the beneficial relationship of information-sharing behavior on social and emotional loneliness, we added the variable information-sharing behavior as an independent variable to these baseline models. Second, for testing hypotheses H2a and H2b, i.e., the association between higher number of online contacts and lower social and emotional loneliness, we added the variable online contacts, as an independent variable to the previous models. Third, to test hypotheses H3a and H3b, i.e., the beneficial link of higher interconnectedness of the student’s close contacts and feelings of social and emotional loneliness, we added interconnectedness as an independent variable to the previous models. Fourth, heterogeneity of the close contacts was added to the models as an independent variable to address hypotheses H4a and H4b (i.e., the correlation of lower heterogeneity with lower social and emotional loneliness). Finally, to explore the relative contributions of the predictors to the variance decomposition of the final models, a relative importance analysis was performed using the proportional marginal variance decomposition as proposed by Feldman (2005). Confidence intervals were determined via bootstrapping with *n* = 10,000 bootstrap runs. For the regressions, two-sided hypothesis tests were used, each with an alpha level of α = 0.05.

For the mentioned analyses, we utilized R (v4.0.4; R Core Team, 2020), as well as the following packages: for general descriptives *psych v2.1.3* (Revelle, 2020), for graphics *ggplot2 v3.3.3* and *scatterplot3d v0.3-41* (Ligges and Mächler, 2003; Wickham, 2016), for cluster analysis *factoextra v1.0.7* (Kassambara and Mundt, 2020), for t-SNE analysis *Rtsne v0.15* (Krijthe, 2015), for calculating Shannon entropy *vegan v2.5-7* (Oksanen et al., 2020), and for relative importance analysis *relaimpo* v2.2-5 (Grömping, 2006).

## Results

### Descriptives and Correlations

The descriptives of the participants included in the analysis can be found in Table 1. Students had—on average—an equal number of offline and online contacts, *t*(558.01) = 0.63, *p* = 0.53. Fifty-eight students (i.e., 20%) reported the maximum of eight possible close contacts. Every second student (i.e., 54%) reported having communicated with their significant other (i.e., partner). The value of mean interconnectedness (i.e., a density value of 0.68) indicates that approximately two-thirds of all possible acquaintance connections between the students’ contacts were present, which is considered as high (Giannella and Fischer, 2016). Additional results can be found in Table 1.

The correlation analysis, see Table 2, showed—as expected—a high correlation between emotional and social loneliness. Social loneliness correlated higher with the number of contacts (online as well as offline) and digital information-sharing behavior than did emotional loneliness. The number of close contacts (i.e., sum of offline and online contacts) correlated moderately with social loneliness, *r*(281) = –0.36, *p* < 0.001, and weakly with emotional loneliness, *r*(281) = –0.12, *p* = 0.047.

**TABLE 2:** Pearson’s correlation coefficients of all variables of interest (*n* = 283).

	1.	2.	3.	4.	5.	6.	7.	8.	9.	10.
1. Gender		–0.11	**–0.13**	0.01	0.03	**0.15**	–0.06	0.11	**0.15**	0.07
2. Age			–0.02	**–0.14**	–0.01	**0.13**	0.06	0.12	–0.02	0.10
3. Social loneliness				**0.56**	**–0.19**	–0.09	**–0.19**	**–0.20**	–0.08	–0.07
4. Emotional loneliness			**0.57**		–0.05	–0.07	**–0.12**	–0.08	–0.03	0.07
5. Offline contacts			**–0.19**	–0.06		0.11	0.01	**–0.38**	0.01	**0.32**
6. Partner			–0.07	–0.05	0.11		**0.27**	–0.05	**0.25**	0.10
7. Inf.-sharing beh.			**–0.20**	–0.11	0.02	**0.28**		**0.12**	–0.05	–0.02
8. Online contacts			**–0.19**	–0.06	**–0.39**	–0.09	**0.12**		**–0.29**	**0.21**
9. Interconnectedness			–0.06	–0.03	<0.01	**0.23**	–0.04	**–0.32**		0.11
10. Heterogeneity			–0.06	0.09	**0.32**	0.08	–0.02	**0.19**	0.10	

*The upper triangular matrix represents Pearson’s bivariate correlation coefficients; the lower triangular matrix shows partial bivariate correlation coefficients controlled for age and gender. Correlation coefficients with p < 0.05 are marked bold. No alpha error cumulation correction was applied. Inf.-sharing beh. stands for information-sharing behavior.*

### Social Loneliness

#### Regression Results

To assess the associations between social loneliness and the assumed independent variables, a hierarchical linear regression was conducted. All results can be found in Table 3. All resulting linear regression models showed good fit, indicated by normally distributed residual variances and no signs of heteroscedasticity. The variance inflation factors (O’Brien, 2007), and the condition numbers (Kim, 2019) of all models indicated no collinearity between the predictors.

**TABLE 3:** Regression results for social loneliness as the criterion.

Predictor	Base model	Model 1	Model 2	Model 3	Model 4
Gender	−0.12*	−0.14*	–0.09	–0.06	–0.05
Age	–0.02	–0.02	0.02	0.02	0.01
Offline contacts	−0.18**	−0.19**	−0.30**	−0.33**	−0.41**
Partner	–0.05	0.01	–0.02	0.02	0.02
Inf.-sharing beh.		−0.20**	−0.16**	−0.17**	−0.15**
Online contacts			−0.29**	−0.36**	−0.44**
Interconnectedness				−0.18**	−0.22**
Heterogeneity					0.17**
*R* ^2^	0.055	0.092	0.159	0.186	0.208
Δ*R*^2^	0.055**	0.037**	0.067**	0.027**	0.021**

Standardized regression coefficients. A more detailed table is depicted in Supplementary Appendix. *Indicates p < 0.05. ** indicates p < 0.01. Inf.-sharing beh. stands for information-sharing behavior.

Starting from a base model, we added the appropriate predictors for each hypothesis. The base model including gender, age, offline contacts and presence of partner showed a fit of adjusted *R*^2^ = 0.042, *F*_(4, 278)_ = 4.07, *p* = 0.003; see Table 3 for more details. While a higher number of offline contacts was associated with decreased social loneliness (β = –0.18, *p* = 0.002), the presence of a partner showed no significant effect. While age was not related to loneliness, female participants showed higher levels of loneliness on average, β = –0.12, *p* = 0.049.

By adding information-sharing behavior as a predictor, the model fit improved significantly by Δ*R*^2^ = 0.037, *p* = 0.009, as displayed in Table 3. The significant regression coefficient indicated a relationship between increased information-sharing behavior and decreased social loneliness (β = –0.20, *p* < 0.001). Thus, we accept Hypothesis 1a.

The number of online contacts increased the model fit significantly by Δ*R*^2^ = 0.067, *p* < 0.001 and showed a significant negative association with social loneliness (β = –0.29, *p* < 0.001), as displayed in Table 3, indicating that having more online contacts is associated with less social loneliness. Thus, we accept Hypothesis 2a.

By adding the interconnectedness of the students’ contacts, the model fit significantly increased by Δ*R*^2^ = 0.027, *p* = 0.003. The significant negative regression weight indicated that an increase of interconnectedness was associated with lower social loneliness (β = –0.18, *p* = 0.001). For more details (see Table 3). Thus, we accept Hypothesis 3a.

In the final step of the hierarchical regression, the added heterogeneity predictor significantly increased the model by Δ*R*^2^ = 0.021, *p* = 0.007, thus resulting in a final model fit of adjusted *R*^2^ = 0.18. As expected, a lower heterogeneity (i.e., higher homogeneity) was significantly associated with lower social loneliness (β = 0.17, *p* = 0.003, see Table 3). This final model indicates that having many contacts of a predominately few types or not uniformly distributed types is associated with low social loneliness. Thus, we accept Hypothesis 4a.

#### Relative Importance Analysis of the Final Model

To assess the various contributions to explained variance, we conducted a relative importance analysis of the final step of the linear regression model. The results can be found in Table 4. The final model explained *R*^2^ = 0.21 of total variance regarding social loneliness as the criterion, of which age and gender explained *R*^2^ = 0.02. Of the remaining *R*^2^ = 0.19 variance, digital information-sharing behavior explained 14.8% (i.e., *R*^2^ = 0.03 of total variance). Both offline and online contacts were the most relevant contributors to the explained variance; together they resulted in approximately two thirds of *R*^2^ (i.e., 64.8%, or *R*^2^ = 0.12 of total variance, respectively). Offline and online contacts did not differ in their amount of explained variance, indicating that the communication channel with close contacts shows no differences regarding the association with social loneliness. The two predictors—interconnectedness and heterogeneity—contributed 12.3% (i.e., *R*^2^ = 0.02) and 8% (i.e., *R*^2^ = 0.02) respectively, to *R*^2^.

**TABLE 4:** Relative importance analysis results.

Predictors	Absolute variance explained	Proportion of variance explained	Lower 95% CI	Upper 95% CI	Significant differences
Offline contacts	0.06	32.25	18.08	46.94	a, c
Partner	<0.01	0.15	0.00	7.96	a, b
Inf.-sharing beh.	0.03	14.84	1.83	36.09	
Online contacts	0.06	32.43	17.15	48.22	b, d
Interconnectedness	0.02	12.32	3.26	25.10	
Heterogeneity	0.02	8.00	1.03	16.43	c, d

*Explained variances of included predictors for social loneliness as the criterion. R^2^ = 0.21, of which 0.02 is explained by gender and age. Significant differences (p < 0.05) in explained variance are marked with the same letter (e.g., the proportions of variance explained of offline contacts and partner differ significantly, indicated by the same letter “a”). The confidence intervals (CI) might be inflated (Grömping, 2006). Inf.-sharing beh. stands for information-sharing behavior.*

### Emotional Loneliness

#### Regression Results

To assess the associations between emotional loneliness and the predictors, a hierarchical linear regression analysis was conducted. All results can be found in Table 5. All resulting linear regression models showed good fit, indicated by normally distributed residual variances and no signs of heteroscedasticity. The variance inflation factors (O’Brien, 2007), and the condition numbers (Kim, 2019) of all models indicated no collinearity between the predictors.

**TABLE 5:** Regression results using emotional loneliness as the criterion.

Predictor	Base model	Model 1	Model 2	Model 3	Model 4
Gender	0.00	–0.01	0.01	0.02	0.03
Age	−0.14*	−0.14*	−0.13*	−0.12*	−0.13*
Offline contacts	–0.05	–0.05	–0.08	–0.09	−0.19**
Partner	–0.05	–0.02	–0.02	–0.01	–0.01
Inf.-sharing beh.		–0.11	–0.10	–0.10	–0.08
Online contacts			–0.08	–0.11	−0.20**
Interconnectedness				–0.06	–0.11
Heterogeneity					0.20**
*R* ^2^	0.026	0.037	0.043	0.046	0.074
Δ*R*^2^	0.026*	0.011	0.006	0.003	0.028**

Standardized regression coefficients. A more detailed table can be found in Supplementary Appendix. * Indicates p < 0.05. ** indicates p < 0.01. Inf.-sharing beh. stands for information-sharing behavior.

Starting from a baseline model, we added the appropriate predictors for each hypothesis. The baseline model, including gender, age, offline contacts, and presence of partner, showed a fit of adjusted *R*^2^ = 0.01. Neither a higher number of offline contacts nor the presence of a partner showed any significant effect on emotional loneliness. Solely higher age was associated with lower loneliness experience (β = –0.14, *p* = 0.023).

No significant association between digital information-sharing behavior and emotional loneliness was found (β = –0.11, *p* = 0.078). For more details (see Table 5). Thus, we reject Hypothesis 1b.

No significant association between the number of online contacts and emotional loneliness was found (β = –0.05, *p* = 0.101), thus we rejected Hypothesis 2b.

No significant association between the interconnectedness of the students’ contacts and emotional loneliness was found (β = −0.06, *p* = 0.164), thus we rejected Hypothesis 3b.

By adding heterogeneity of the students’ contacts, the model fit increased by Δ*R*^2^ = 0.028, thus resulting in a final model fit of adjusted *R*^2^ = 0.05. A significant positive association between the heterogeneity of students’ contacts and emotional loneliness was found (β = 0.20, *p* = 0.002), as displayed in Table 5, indicating homogenous networks are associated with lower emotional loneliness. By adding heterogeneity, the two predictors offline and online contacts turned significant which is addressed in more detail in the discussion. Thus, we accept Hypothesis 4b.

#### Relative Importance Analysis of the Final Model

To assess the various contributions to explained variance, we conducted a relative importance analysis of the final step of the linear regression model. The results can be found in Table 6. The final model explained *R*^2^ = 0.07 of total variance regarding emotional loneliness as the criterion, of which age and gender explained *R*^2^ = 0.02 of the remaining *R*^2^ = 0.05 variance, digital information-sharing behavior explained 18.9% (i.e., *R*^2^ = 0.01 of total variance). Both offline and online contacts were the large contributors to the explained variance, added together they resulted in approximately 42.5% of *R*^2^ (i.e., *R*^2^ = 0.02 of total variance). They did not differ in their amount of explained variance. Interconnectedness contributed 12.0% to *R*^2^. Heterogeneity made up approximately one quarter of *R*^2^ (i.e., 26.2% or *R*^2^ = 0.01 of total variance).

**TABLE 6:** Relative importance analysis results.

Predictors	Absolute variance explained	Proportion of variance explained in %	Lower 95% CI	Upper 95% CI	Significant differences
Offline contacts	0.01	21.43	3.26	47.26	
Partner	<0.01	0.47	0.01	33.82	
Inf.-sharing beh.	0.01	18.88	0.05	54.82	
Online contacts	0.01	21.10	2.61	46.03	
Interconnectedness	0.01	11.95	0.14	32.73	
Heterogeneity	0.01	26.17	4.45	53.71	

*Explained variances of included predictors for emotional loneliness as the criterion. R^2^ = 0.07, of which 0.02 is explained by gender and age. Significant differences (p < 0.05) in explained variance are marked with the same letter (i.e., there are no significant differences). The confidence intervals (CI) might be inflated (Grömping, 2006). Inf.-sharing beh. stands for information-sharing behavior.*

## Discussion

In this study, we investigated higher education students’ perceptions of loneliness in a German sample. The COVID-19 contact restrictions and the rapid move to emergency remote teaching in higher education resulted in a loss of in-person contact. In this context, we examined how digital information-sharing behavior and the structure of students’ close network (i.e., number of close contacts with whom they communicated predominately online or offline, interconnectivity of close contacts, and heterogeneity of close contacts) were related to students’ feelings of loneliness. Here, we examined social and emotional loneliness separately. We performed hierarchical linear regressions and examined the predictive strength of the predictors via relative importance analyses.

In summary, our findings indicate that social loneliness is strongly related to digital information-sharing behavior and the network structure of close contacts. In particular, high information-sharing behavior, many close contacts (regardless of whether offline or online), a highly interconnected network, and a homogeneous structure of close contacts were associated with low social loneliness. Emotional loneliness, on the other hand, was mainly linked with network homogeneity, in the sense that students with homogeneous networks showed low emotional loneliness.

Regarding our first hypothesis, we looked at the relationships between information-sharing behavior and social and emotional loneliness. Information-sharing behavior showed a favorable relationship with social loneliness: Students with higher information-sharing behavior showed lower social loneliness perceptions. We could not find a significant association between information-sharing behavior and emotional loneliness. Several possible explanations exist for this connection. The imposed COVID-19 restriction led to two relevant changes in students’ lives. First, there was a switch to emergency remote teaching, and second, face-to-face contact was reduced. Emergency remote teaching posed stressful challenges for many students and instructors (Clabaugh et al., 2021). Digital information-sharing behavior facilitated the use of the new focus on remote teaching (Bergdahl et al., 2020). Students who were able to cope well with the new virtual learning environment therefore experienced less stress, which enables them to experience less feelings of loneliness (Yarcheski et al., 2011; Händel et al., 2020). Information-sharing behavior also seemed to be helpful outside the higher education learning context. Our correlation analysis results imply that higher digital information-sharing behavior facilitates staying in touch with a higher number of close contacts, and thus might be linked with decreased feelings of social loneliness (as suggested by Hypothesis 2). The non-significant association between information-sharing behavior and emotional loneliness may be explained by the nature of the Covid-19 restriction in Germany. Although personal contacts were severely restricted at the height of the restrictions, it was still possible to meet another person from another household in addition to people from one’s own household. Therefore, participants were able to meet their most important social contact, usually their own partner or best friend, resulting in little or no need to shift communication to online communication. Further—preferably longitudinal—research should explore this question in more detail.

In our second hypothesis, we tested the relationship between close contacts with whom mainly online communication took place and social as well as emotional loneliness. In the case of social loneliness, we found a relationship in accordance with the assumptions, i.e., the more online contacts, the less lonely. In the case of emotional loneliness, this relationship only emerged in the final model, considering all subsequent effects. As other research suggests (Subrahmanyam and Greenfield, 2008; Reich et al., 2012), we assume that due to involuntary contact termination by the COVID-19 restrictions, communication with offline contacts was inevitably shifted to online communication. In line with our results, this means that in the case of social loneliness, which according to the definition is mainly related to the number of close contacts, online communication could—possibly only temporarily—act as a substitute for the lack of in-person, face-to-face exchange. However, the desire for intimacy is not associated with many close friends, resulting in weaker associations with emotional loneliness (Russell et al., 1984). This is also supported by our relative importance analysis where the absolute proportion of variance of close contacts (no matter whether offline or online) was lower for emotional loneliness than it was for social loneliness. This result suggests that it is not the number of close contacts that is decisive, but rather, as mentioned for example by Weiss (1973), the quality of certain few contacts is significant toward (not) developing feelings of emotional loneliness.

Our third hypothesis tested whether higher levels of close contact interconnectedness were associated with lower levels of loneliness. Our results indicate that increased interconnectivity was associated with lower social, but not emotional, loneliness. Interconnectedness can derive from an evolutionary mechanism of social networks, namely triadic closure (Schaefer et al., 2010; Bianconi et al., 2014), i.e., if a person has two close friends, the two friends will almost inevitably get to know each other over time (e.g., through shared activities, or a shared social environment). Over time, this leads to an interconnected close contacts network. For many social networks, a high level of interconnectivity indicates functioning social support, since the network consists of people who know each other and thus originating from the same social environment (Jones and Moore, 1989; Ashida and Heaney, 2008). Both interconnectedness and social support have been shown to have favorable impacts on feelings of loneliness (Jones and Moore, 1989; Bell, 1991; Ashida and Heaney, 2008). The unobserved effect of interconnectedness on emotional loneliness in our study could probably be due to the assumption that emotional loneliness is mainly related to significant others (e.g., life partners, Russell et al., 1984). Significant others usually account for only a small proportion of the close social network (Dunbar, 1998; Zhou et al., 2005) and therefore, they exert relatively little impact on interconnectivity (i.e., the measure of network density).

In our fourth hypothesis, we considered the relationship between heterogeneity of the students’ close contacts and feelings of loneliness; we hypothesized that higher homogeneity would be associated with lower feelings of social and emotional loneliness. To derive the heterogeneity, we applied the Shannon entropy of the close contact types, which we obtained by using a combination of t-distributed stochastic neighbor embedding and cluster analysis. The final hierarchical linear regression model demonstrated that higher contact homogeneity was associated with lower social and emotional loneliness. High homogeneity is often associated with networks of trusted people, who provide social support that might allow students to better cope with new challenges (Salehi et al., 2019; Simons et al., 2020). Shannon entropy as a measure of heterogeneity allows two conclusions about the structure of these social support networks. A high degree of homogeneity (i.e., low heterogeneity) can indicate (1) a low number of types or (2) a non-uniform distribution of the present contact types in the social support network. In other words, the support network structure consists either of a few contact types (e.g., four persons of “type 1” and four persons of “type 2”) or of several types that are non-uniformly distributed (i.e., some types are disproportionately frequent, e.g., six persons of “type 1,” one person of “type 2,” and one person of “type 3”). It is possible to determine which of the two possibilities applies by considering the other two predictors relating to the number of close contacts of the multiple regression (i.e., number of offline and online contacts). Because the number of online and offline contacts is correlated with the number of types (*r* ≈ 0.6), the additional explained variance of the loneliness measures due to heterogeneity in our hierarchical model can be attributed to the two structural possibilities by examining the regression weights of online and offline contacts *before* and *after* adding heterogeneity to the model.

In the case of social loneliness, both predictors online and offline contacts were already significantly associated with social loneliness before the inclusion of our heterogeneity measure. We assume that the additional explained variance after adding heterogeneity to our model consequently indicates homogeneity due to a low number of types than a non-uniform distribution of types. However, the latter possibility (i.e., non-uniformly distributed types) cannot be completely excluded due to the increased regression weights of online and offline contacts after adding heterogeneity to the model. Either way, according to our results, social support networks associated with low social loneliness are characterized by many contacts with a low effective number of types (either few types, or individual types are heavily overrepresented).

In the case of emotional loneliness, probably only one structural property of these social support networks applies: the non-uniformity of types, i.e., an overrepresentation of individual types. By adding the predictor heterogeneity, the originally non-significant predictors online and offline contacts turned significant, indicating shared variance between heterogeneity and the predictors. Since the number of types is correlated with the number of online and offline contacts, the remaining residual effect of heterogeneity mainly describes the distribution of types. Consequently, in the case of emotional loneliness, an overrepresentation of a few individual types (i.e., the types are non-uniformly distributed, as seen in social support networks, Coffé and Geys, 2007) is associated with lower feelings of loneliness.

Overall, regarding social loneliness, we interpret our network related observations as follows: we conclude that close contact networks formed according to principles of selection, influence and linkage formation and thus, consisting of many individuals of mainly a few types, are associated with lower feelings of social loneliness. In the case of emotional loneliness, we assume that a different explanatory possibility applies—mainly specific types (e.g., significant others) of the close contacts network might be associated with lower emotional loneliness. Therefore, the absolute number of close contacts might be less important in this context, but rather a prioritization of these special types.

### Limitations, Future Research, and Implications

We would like to address some limitations of our study and simultaneously put the interpretation of the results in context. First, since the present study consists of a cross-sectional analysis, these results only represent correlational associations. However, a comparison of longitudinal results with cross-sectional results in similar research contexts indicates that they may well be very comparable (Newall et al., 2009). Nevertheless, further research should shed more light on the dynamic nature of the interactions found. For example, longitudinal analyses could additionally consider network development and explore the interplay between selection, influence, and feelings of loneliness. Can contagion processes regarding feelings of loneliness be demonstrated again, as indicated by Cacioppo et al. (2009)? What is the temporal pattern of our observed correlational effects?

Second, we would like to address our sample. It showed a gender bias and mainly was situated in one state and one university; therefore, there might be limited generalizability to populations outside those represented by our sample. Regarding the gender bias, a comparison with the validation analysis of the utilized loneliness scales suggests that our results might be representative (Jong Gierveld and Van Tilburg, 2010). In addition, our results indicate that age is related to emotional loneliness, and younger adults report higher emotional loneliness than older adults do. This is consistent with previous research (Yang and Victor, 2011; Ang, 2016; Nyqvist et al., 2016) and may support the generalizability of our sample. In addition, we cannot rule out the possibility of an overlap of egocentric networks (e.g., through shared circles of friends or living in the same apartment) that would violate the necessary statistical independence for linear regression. However, we expect a negligible influence on our analyses since students attended different faculties and a large proportion of students lived with their parents rather than at the university during the COVID-19 restrictions. Two other points worth mentioning are the relatively small sample size and the convenience sample. Both aspects could explain why we did not find a significant correlation between the presence of a partner and (emotional) loneliness. Overall, we note that while our sample is likely to have limited representativeness, it nevertheless provides an interesting initial insight into students’ feelings of loneliness during a pandemic period when only people from a maximum of two different households were allowed to meet. Still, there is a need to repeat our analyses with a larger, randomized sample to obtain more reliable statistical results.

Third, in our study, we wanted to advance a networks heterogeneity measure one step further by employing Shannon entropy. There are two reasons why we would like to emphasize that our study may have limited comparability with previous research in terms of heterogeneity. First, our method of measuring heterogeneity, and second, the context in which previous research has viewed heterogeneity and loneliness. First, to our knowledge, our study is the first to use the heterogeneity measure entropy in students’ close networks. Other studies often employ the so-called social network index, which counts only the number of social types (e.g., spouse, parents, friends, etc.) with which an individual interacts (Robustelli et al., 2017; Liebke, 2019). However, when measuring heterogeneity or homogeneity, respectively, both the distribution of types and a sophisticated type classification method should be used (Jost, 2006). We addressed this shortcoming through a more sophisticated type assignment and the use of Shannon entropy. It is often used in ecology, information theory, and thermodynamics (Jost, 2006; Masisi et al., 2008); we encourage its use in a psychological research context as well. Second, the context of our study is in part, very distinct from that of earlier research. Our data reflected only a rather short-term impact of the COVID-19 restrictions on students’ feelings of loneliness. Most students were aware—or at least hoped—that these restrictions were only a temporary solution. Furthermore, it should be noted that a considerable part of public and private life was affected by the COVID-19 measures and therefore, it is likely that each close contact in the network experienced impacts on their loneliness. Indeed, previous loneliness research had been conducted in a different context, and with different populations. Populations heretofore studied in loneliness research were often challenged by a lack of social support in their individual social networks (e.g., elderly, Fry and Debats, 2002; as well as populations with mental health challenges, such as, borderline personality disorder, Liebke, 2019), where heterogeneity proves beneficial to lower feelings of loneliness due to its link to resilience (Elmqvist et al., 2003). Considering the aforementioned points (i.e., our method of measuring heterogeneity and our distinctive study context), our results on the heterogeneity of close contacts networks have limited comparability with previous research. Moreover, although the entropy measure represents an advance over previous heterogeneity measures in network research, we encourage future research to compare other indicators of heterogeneity or homogeneity in individual network environments, or even explore ensemble statistics of multiple classifiers (Masisi et al., 2008).

Fourth, the relatively low effect sizes for emotional loneliness could also be associated with the acceptable but rather low omega hierarchical. A value of ω_h_ = 0.66 indicates a rather poor unidimensional construct, which might affect our results. This could also be a reason why the presence of a partner showed only a non-significant value in the regression analysis. Additional, research findings that a partner is less predictive of emotional loneliness in young adults than in older adults may play a role here (Green et al., 2001). In addition, a relatively high number of students (i.e., 20%) reported the maximum possible number of close contacts (i.e., eight), indicating a ceiling effect. While this had no discernible impact on our results, we recommend that, if the scope of the study allows it, future research should provide twelve or more fields for recording close contacts to better account for the skewed distribution. Moreover, the observed results for emotional loneliness open up another possible interpretation: there may be an unrecognized positive relationship between the number of contacts and emotional loneliness after all (i.e., increased close contacts and decreased emotional loneliness, see Hypothesis 2b). The effect of the number of close contacts could be masked by its association with the number of types and thus with heterogeneity in model 3 (Table 5) in the following ways: A higher number of close contacts is correlated with a higher number of types present and thus with higher heterogeneity, which is negatively related to loneliness, thus negating the “positive” effect of the number of contacts. Future research should investigate this possible relationship in more detail.

From our results, first indications for practical implications can be derived—albeit to a limited extent due to the limitations mentioned above. In order to strengthen the positive influence of information-sharing behavior, universities should rely on easy-to-use communication software and offer trainings on their optimal use for lecturers as well as students. In this way, not only the quality of online teaching might increase but the hurdle for students to communicate with their peers is also kept low. Our research revealed a strong association of students’ close social network, which consisted largely of peers (see Supplementary Appendix), with feelings of loneliness. Other research reinforces this connection through highlighting the importance of perceived peer support on feelings of loneliness (e.g., Kaufmann and Vallade, 2020; Laslo-Roth et al., 2020). Here the lecturer plays a central role in facilitating these beneficial effects (Kaufmann and Vallade, 2020). In online teaching, the lecturer should try to promote interactions between students and their peers (e.g., through group work in breakout rooms), to provide opportunities for the development of online peer support relationships (Kaufmann and Vallade, 2020). It should be noted that such a supportive environment is more likely to be created by synchronous teaching methods (e.g., via video chat platform) and therefore the exclusive use of pre-recorded material should be avoided. Moreover, our results imply that interventions for loneliness (outside the context of higher education) should not only take into account the individual but also the individual’s network. Here, the focus could lie on connecting and expanding the close network (ongoing research projects are already being conducted, e.g., Band et al., 2019).

## Conclusion

The present study provides valuable information about how students’ close contacts network structures and digital information-sharing behavior are linked to their experience of loneliness in the context of the COVID-19 pandemic. This study distinguished between social (related to the number of contacts) and emotional (related to intimate contacts) loneliness and showed different associations with the investigated predictors. Overall, social loneliness was more strongly associated with the network structure of close contacts than was emotional loneliness. A higher number of close contacts, high interconnectivity and strong homogeneity of those networks were associated with lower feelings of loneliness—more with social than with emotional loneliness—regardless of whether the communication between the student and their close contacts took place primarily online or offline. We concluded that homogeneous network structures, which are an indicator of social support networks, were linked with lower feelings of loneliness. In addition, digital information-sharing behavior, which might have facilitated transfer from offline to online communication, was found to help students cope with feelings of social loneliness. This study demonstrates that a functioning close social network and suitable usage of digital tools are important to cope with new social and educational environments that will continue to play a decisive role in students’ lives, even after COVID-19.

## Data Availability Statement

The raw data supporting the conclusions of this article will be made available by the authors, without undue reservation.

## Ethics Statement

Ethical review and approval was not required for the study on human participants in accordance with the local legislation and institutional requirements. Written informed consent for participation was not required for this study in accordance with the national legislation and the institutional requirements.

## Author Contributions

MDSH, AZ, and MH developed the research idea and developed the questionnaire. MDSH and MH processed the data. MDSH performed the statistical analysis and wrote the manuscript. All authors provided feedback, participated in revising the manuscript, reviewed, and approved the final manuscript.

## Conflict of Interest

The authors declare that the research was conducted in the absence of any commercial or financial relationships that could be construed as a potential conflict of interest.

## Publisher’s Note

All claims expressed in this article are solely those of the authors and do not necessarily represent those of their affiliated organizations, or those of the publisher, the editors and the reviewers. Any product that may be evaluated in this article, or claim that may be made by its manufacturer, is not guaranteed or endorsed by the publisher.

## Footnotes

1 Other measures such as the Blau index (also known as Gibbs-Martin index or Gini-Simpson index) represent a similar reliable measurement of heterogeneity (Jost, 2006) and showed no major changes in our results.

## References

[B1] Al Khatib S. A. (2012). Exploring the Relationship among Loneliness, Self-esteem, Self-efficacy and Gender in United Arab Emirates College Students. *Eur. J. Psychol.* 8:1. 10.5964/ejop.v8i1.301 33680184

[B2] Alkan N. (2014). Humor, Loneliness and Acceptance: predictors of University Drop-out Intentions. *Procedia Soc. Behav. Sci.* 152 1079–1086. 10.1016/j.sbspro.2014.09.278

[B3] Ang C.-S. (2016). Types of Social Connectedness and Loneliness: the Joint Moderating Effects of Age and Gender. *Appl. Res. Qual. Life* 11 1173–1187. 10.1007/s11482-015-9428-5

[B4] Ashida S. Heaney C. A. (2008). Differential Associations of Social Support and Social Connectedness With Structural Features of Social Networks and the Health Status of Older Adults. *J. Aging Health* 20 872–893. 10.1177/0898264308324626 18815414

[B5] Band R. Ewings S. Cheetham-Blake T. Ellis J. Breheny K. Vassilev I. (2019). Study protocol for ‘The Project About Loneliness and Social networks (PALS)’: a pragmatic, randomised trial comparing a facilitated social network intervention (Genie) with a wait-list control for lonely and socially isolated people. *BMJ Open* 9:e028718. 10.1136/bmjopen-2018-028718 31427326 PMC6701612

[B6] Bell R. A. (1991). Gender, Friendship Network Density, and Loneliness. *J. Soc. Behav. Pers.* 6 45–56.

[B7] Benkel I. Wijk H. Molander U. (2009). Family and friends provide most social support for the bereaved. *Palliat. Med.* 23 141–149. 10.1177/0269216308098798 18952747

[B8] Bergdahl N. Nouri J. Fors U. (2020). Disengagement, engagement and digital skills in technology-enhanced learning. *Educ. Inf. Technol.* 25 957–983. 10.1007/s10639-019-09998-w

[B9] Bernholt A. Hagenauer G. Lohbeck A. Gläser-Zikuda, Michaela Wolf N. Moschner B. (2018). Bedingungsfaktoren der Studienzufriedenheit von Lehramtsstudierenden. *J. Educ. Res. Online* 10 22–49.

[B10] Bianconi G. Darst R. K. Iacovacci J. Fortunato S. (2014). Triadic closure as a basic generating mechanism of communities in complex networks. *Phys. Rev.* 90:042806. 10.1103/PhysRevE.90.042806 25375548

[B11] Bu F. Steptoe A. Fancourt D. (2020). Who is lonely in lockdown? Cross-cohort analyses of predictors of loneliness before and during the COVID-19 pandemic. *Public Health* 186 31–34. 10.1016/j.puhe.2020.06.036 32768621 PMC7405905

[B12] Burk W. J. Kerr M. Stattin H. (2008). The co-evolution of early adolescent friendship networks, school involvement, and delinquent behaviors. *Rev. Française Sociol.* 49:499. 10.3917/rfs.493.0499 18052372

[B13] Cacioppo J. T. Fowler J. H. Christakis N. A. (2009). Alone in the crowd: the structure and spread of loneliness in a large social network. *J. Pers. Soc. Psychol.* 97 977–991. 10.1037/a0016076 19968414 PMC2792572

[B14] Caravita S. C. S. Sijtsema J. J. Rambaran J. A. Gini G. (2014). Peer influences on moral disengagement in late childhood and early adolescence. *J. Youth Adolesc.* 43 193–207. 10.1007/s10964-013-9953-1 23660831

[B15] Clabaugh A. Duque J. F. Fields L. J. (2021). Academic Stress and Emotional Well-Being in United States College Students Following Onset of the COVID-19 Pandemic. *Front. Psychol.* 12:628787. 10.3389/fpsyg.2021.628787 33815214 PMC8010317

[B16] Coffé H. Geys B. (2007). Toward an Empirical Characterization of Bridging and Bonding Social Capital. *Nonprofit Volunt. Sect. Q.* 36 121–139. 10.1177/0899764006293181

[B17] Dai P. Wang N. Kong L. Dong X. Tian L. (2021). A large number of online friends and a high frequency of social interaction compensate for each Other’s shortage in regard to perceived social support. *Curr. Psychol.* 6 1–10. 10.1007/s12144-021-01458-4 33716471 PMC7936227

[B18] de Jong Gierveld J. Van Tilburg T. (1999). *Manual of the Loneliness Scale.* Amsterdam: Departement of Social Research Methodology, Vrije Universiteit.

[B19] Dunbar R. I. M. (1998). The social brain hypothesis. *Evol. Anthropol.* 6 178–190. 10.1002/(SICI)1520-650519986:5<178::AID-EVAN5<3.0.CO;2-8 29214320

[B20] Dykstra P. A. Jong Gierveld J. D. (2004). Gender and Marital-History Differences in Emotional and Social Loneliness among Dutch Older Adults. *Can. J. Aging* 23 141–155. 10.1353/cja.2004.0018 15334814

[B21] Elmqvist T. Folke C. Nystrom M. Peterson G. Bengtsson J. Walker B. (2003). Response Diversity, Ecosystem Change, and Resilience. *Front. Ecol. Environ.* 1:488. 10.2307/3868116

[B22] Feldman B. E. (2005). *Relative Importance and Value.* 10.2139/ssrn.2255827 Available online at: https://ssrn.com/abstract=2255827 (accessed June 20, 2021).

[B23] Fry P. S. Debats D. L. (2002). Self-Efficacy Beliefs as Predictors of Loneliness and Psychological Distress in Older Adults. *Int. J. Aging Hum. Dev.* 55 233–269. 10.2190/KBVP-L2TE-2ERY-BH26 12693547

[B24] Giannella E. Fischer C. S. (2016). An inductive typology of egocentric networks. *Soc. Netw.* 47 15–23. 10.1016/j.socnet.2016.02.003

[B25] Granovetter M. S. (1973). The Strength of Weak Ties. *Am. J. Sociol.* 78 1360–1380. 10.1086/225469

[B26] Green L. R. Richardson D. S. Lago T. Schatten-Jones E. C. (2001). Network Correlates of Social and Emotional Loneliness in Young and Older Adults. *Personal. Soc. Psychol. Bull.* 27 281–288. 10.1177/0146167201273002

[B27] Grömping U. (2006). Relative Importance for Linear Regression in R: the Package relaimpo. *J. Stat. Softw.* 17 1–27. 10.18637/jss.v017.i01

[B28] Händel M. Stephan M. Gläser-Zikuda M. Kopp B. Bedenlier S. Ziegler A. (2020). *Digital Readiness and its Effects on Higher Education Students’ Socio-Emotional Perceptions in the Context of the COVID-19 Pandemic.* United Kingdom: Taylor & Francis. 1–13. 10.1080/15391523.2020.1846147

[B29] Hodges C. Moore S. Lockee B. Trust T. Bond A. (2020). *The Difference Between Emergency Remote Teaching and Online Learning.* Available online at: https://er.educause.edu/articles/2020/3/the-difference-between-emergency-remote-teaching-and-online-learning (accessed June 16, 2021).

[B30] Hofstra B. Corten R. van Tubergen F. Ellison N. B. (2017). Sources of Segregation in Social Networks: a Novel Approach Using Facebook. *Am. Sociol. Rev.* 82 625–656. 10.1177/0003122417705656

[B31] Hong A. J. Kim H. J. (2018). College Students’ Digital Readiness for Academic Engagement (DRAE) Scale: scale Development and Validation. *Asia-Pacific Educ. Res.* 27 303–312. 10.1007/s40299-018-0387-0

[B32] Honicke T. Broadbent J. (2016). The influence of academic self-efficacy on academic performance: a systematic review. *Educ. Res. Rev.* 17 63–84. 10.1016/j.edurev.2015.11.002

[B33] Hopp M. D. S. Stoeger H. Ziegler A. (2020). The Supporting Role of Mentees’ Peers in Online Mentoring: a Longitudinal Social Network Analysis of Peer Influence. *Front. Psychol.* 11:1929. 10.3389/fpsyg.2020.01929 32922332 PMC7456988

[B34] Hopp M. D. S. Zhang Z. S. Hinch L. O’Reilly C. Ziegler A. (2019). Creative, Thus Connected: the Power of Sociometric Creativity on Friendship Formation in Gifted Adolescents—A Longitudinal Network Analysis of Gifted Students. *New Dir. Child Adolesc. Dev.* 2019 47–73. 10.1002/cad.20324 31702108

[B35] IBM Corp (2019). *IBM SPSS Statistics for Windows, Version 26.0.* Armonk, NY: IBM Corp.

[B36] Jackson T. Soderlind A. Weiss K. E. (2000). Personality traits and quality of relationships as predictors of future loneliness among American college students. *Soc. Behav. Pers. Int. J.* 28 463–470. 10.2224/sbp.2000.28.5.463

[B37] Jones W. H. Moore T. L. (1989). “Loneliness and Social Support” in *Loneliness, Theory, Research and Applications.* eds Hojat M. Crandall R. (United States: SAGE Publications, Inc). 145–156.

[B38] Jong Gierveld J. D. Kamphuls F. (1985). The Development of a Rasch-Type Loneliness Scale. *Appl. Psychol. Meas.* 9 289–299. 10.1177/014662168500900307

[B39] Jong Gierveld D. Van Tilburg T. (2010). The De Jong Gierveld short scales for emotional and social loneliness: tested on data from 7 countries in the UN generations and gender surveys. *Eur. J. Ageing* 7 121–130. 10.1007/s10433-010-0144-6 20730083 PMC2921057

[B40] Jost L. (2006). Entropy and diversity. *Oikos* 113 363–375. 10.1111/j.2006.0030-1299.14714.x

[B41] Kassambara A. Mundt F. (2020). *Factoextra: extract and Visualize the Results of Multivariate Data Analyses.* Available online at: https://cran.r-project.org/package=factoextra (accessed June 20, 2021).

[B42] Kaufmann R. Vallade J. I. (2020). *Exploring Connections in the Online Learning Environment: student Perceptions of Rapport, Climate, and Loneliness.* United Kingdom: Taylor and Francis. 1–15. 10.1080/10494820.2020.1749670

[B43] Killgore W. D. S. Cloonan S. A. Taylor E. C. Dailey N. S. (2020). Loneliness: a signature mental health concern in the era of COVID-19. *Psychiatry Res.* 290:113117. 10.1016/j.psychres.2020.113117 32480121 PMC7255345

[B44] Kim J. H. (2019). Multicollinearity and misleading statistical results. *Korean J. Anesthesiol.* 72 558–569. 10.4097/kja.19087 31304696 PMC6900425

[B45] Kluck J. P. Stoyanova F. Krämer N. C. (2021). Putting the social back into physical distancing: the role of digital connections in a pandemic crisis. *Int. J. Psychol.* 56 594–606. 10.1002/ijop.12746 33615476 PMC8013576

[B46] Kovacs B. Caplan N. Grob S. King M. (2021). *Social Networks and Loneliness During the COVID-19 Pandemic.* United States: Sage Publication. 10.1177/2378023120985254

[B47] Kralj Novak P. Smailović J. Sluban B. Mozetič I. (2015). Sentiment of Emojis. *PLoS One* 10:e0144296. 10.1371/journal.pone.0144296 26641093 PMC4671607

[B48] Kraut R. Kiesler S. Boneva B. Cummings J. Helgeson V. Crawford A. (2002). Internet Paradox Revisited. *J. Soc. Issues* 58 49–74. 10.1111/1540-4560.00248

[B49] Kraut R. Patterson M. Lundmark V. Kiesler S. Mukophadhyay T. Scherlis W. (1998). Internet paradox: a social technology that reduces social involvement and psychological well-being? *Am. Psychol.* 53 1017–1031. 10.1037/0003-066X.53.9.1017 9841579

[B50] Krijthe J. H. (2015). *Rtsne: T-Distributed Stochastic Neighbor Embedding using Barnes-Hut Implementation*. Available online at: https://github.com/jkrijthe/Rtsne (accessed December 21, 2021).

[B51] Laslo-Roth R. Bareket-Bojmel L. Margalit M. (2020). *Loneliness Experience During Distance Learning Among College Students With ADHD: the Mediating Role of Perceived Support and Hope.* United Kingdom: Taylor & Francis. 1–15. 10.1080/08856257.2020.1862339

[B52] Liebke L. (2019). Alterations in Interpersonal Relations in Borderline Personality Disorder: loneliness, Rejection, and Belonging. Available online at: http://www.ub.uni-heidelberg.de/archiv/25987 (accessed June 25, 2021).

[B53] Ligges U. Mächler M. (2003). Scatterplot3d - An R Package for Visualizing Multivariate Data. *J. Stat. Softw.* 8 1–20. 10.18637/jss.v008.i11

[B54] Lin S.-H. Huang Y.-C. (2012). Investigating the relationships between loneliness and learning burnout. *Act. Learn. High. Educ.* 13 231–243. 10.1177/1469787412452983

[B55] Liu S. Heinzel S. Haucke M. N. Heinz A. (2021). Increased Psychological Distress, Loneliness, and Unemployment in the Spread of COVID-19 over 6 Months in Germany. *Medicina* 57:53. 10.3390/medicina57010053 33435368 PMC7827929

[B56] Manago A. M. Brown G. Lawley K. A. Anderson G. (2020). Adolescents’ daily face-to-face and computer-mediated communication: associations with autonomy and closeness to parents and friends. *Dev. Psychol.* 56 153–164. 10.1037/dev0000851 31670551

[B57] Marsden P. V. (1987). Core Discussion Networks of Americans. *Am. Sociol. Rev.* 52:122. 10.2307/2095397

[B58] Masisi L. Nelwamondo V. Marwala T. (2008). “The use of entropy to measure structural diversity” in *2008 IEEE International Conference on Computational Cybernetics* (United States: IEEE). 41–45. 10.1109/ICCCYB.2008.4721376

[B59] Mayer A. Puller S. L. (2008). The old boy (and girl) network: social network formation on university campuses. *J. Public Econ.* 92 329–347. 10.1016/j.jpubeco.2007.09.001

[B60] McDonald R. P. (1999). *Test Theory: a Unified Treatment.* London: Psychology Press.

[B61] McPherson M. Smith-Lovin L. Cook J. M. (2001). Birds of a Feather: homophily in Social Networks. *Annu. Rev. Sociol.* 27 415–444. 10.1146/annurev.soc.27.1.415

[B62] Mercken L. Snijders T. A. B. Steglich C. Vartiainen E. de Vries H. (2010). Dynamics of adolescent friendship networks and smoking behavior. *Soc. Netw.* 32 72–81. 10.1016/j.socnet.2009.02.005 19775794

[B63] Moody E. J. (2001). Internet Use and Its Relationship to Loneliness. *CyberPsychol. Behav.* 4 393–401. 10.1089/109493101300210303 11710265

[B64] Nabi R. L. Prestin A. So J. (2013). Facebook Friends with (Health) Benefits? Exploring Social Network Site Use and Perceptions of Social Support, Stress, and Well-Being. *Cyberpsychology, Behav. Soc. Netw.* 16 721–727. 10.1089/cyber.2012.0521 23790356

[B65] Nakagomi A. Shiba K. Kondo K. Kawachi I. (2020). Can Online Communication Prevent Depression Among Older People? A Longitudinal Analysis. *J. Appl. Gerontol.* 41 167–175. 10.1177/0733464820982147 33356760

[B66] Neto R. Golz N. Polega M. (2015). Social Media Use, Loneliness, and Academic Achievement: a Correlational Study with Urban High School Students. *J. Res. Educ.* 25 28–37.

[B67] Newall N. E. Chipperfield J. G. Clifton R. A. Perry R. P. Swift A. U. Ruthig J. C. (2009). Causal beliefs, social participation, and loneliness among older adults: a longitudinal study. *J. Soc. Pers. Relat.* 26 273–290. 10.1177/0265407509106718

[B68] Nicpon M. F. Huser L. Blanks E. H. Sollenberger S. Befort C. Kurpius S. E. R. (2006). The Relationship of Loneliness and Social Support with College Freshmen’s Academic Performance and Persistence. *J. Coll. Student Retent. Res. Theory Pract.* 8 345–358. 10.2190/A465-356M-7652-783R 22612255

[B69] Nyqvist F. Victor C. R. Forsman A. K. Cattan M. (2016). The association between social capital and loneliness in different age groups: a population-based study in Western Finland. *BMC Public Health* 16:542. 10.1186/s12889-016-3248-x 27400659 PMC4940959

[B70] O’Brien R. M. (2007). A Caution Regarding Rules of Thumb for Variance Inflation Factors. *Qual. Quant.* 41 673–690. 10.1007/s11135-006-9018-6

[B71] Oksanen J. Blanchet F. G. Friendly M. Kindt R. Legendre P. McGlin D. (2020). *vegan: Community Ecology Package.* Available online at: https://cran.r-project.org/package=vegan (accessed June 20, 2021).

[B72] R Core Team (2020). *R: a Language and Environment for Statistical Computing Reference Index.* Vienna. Available online at: http://www.r-project.org/index.html. 10.1109/ICIN.2011.6081064 (accessed June 2, 2021).

[B73] Reich S. M. Subrahmanyam K. Espinoza G. (2012). Friending, IMing, and hanging out face-to-face: overlap in adolescents’ online and offline social networks. *Dev. Psychol.* 48 356–368. 10.1037/a0026980 22369341

[B74] Revelle W. (2020). *psych: Procedures for Psychological, Psychometric, and Personality Research.* Available online at: https://cran.r-project.org/package=psych (accessed January 26, 2021).

[B75] Robustelli B. L. Newberry R. E. Whisman M. A. Mittal V. A. (2017). Social relationships in young adults at ultra high risk for psychosis. *Psychiatry Res.* 247 345–351. 10.1016/j.psychres.2016.12.008 27987484 PMC5217827

[B76] Rosenstreich E. Margalit M. (2015). Loneliness, Mindfulness, and Academic Achievements: a Moderation Effect among First-Year College Students. *Open Psychol. J.* 8 138–145. 10.2174/1874350101508010138

[B77] Rotenberg K. J. Morrison J. (1993). Loneliness and College Achievement: do Loneliness Scale Scores Predict College Drop-Out? *Psychol. Rep.* 73 1283–1288. 10.2466/pr0.1993.73.3f.1283

[B78] Russell D. W. (1982). “The Measurement of Loneliness” in *Loneliness: a Sourcebook of Current Theory, Research and Therapy*, eds Peplau L. Perlman D. (United States: Wiley). 81–104.

[B79] Russell D. W. Cutrona C. E. McRae C. Gomez M. (2012). Is Loneliness the Same as Being Alone? *J. Psychol.* 146 7–22. 10.1080/00223980.2011.589414 22303609

[B80] Russell D. W. Cutrona C. E. Rose J. Yurko K. (1984). Social and emotional loneliness: an examination of Weiss’s typology of loneliness. *J. Pers. Soc. Psychol.* 46 1313–1321. 10.1037/0022-3514.46.6.1313 6737214

[B81] Salehi A. Ehrlich C. Kendall E. Sav A. (2019). Bonding and bridging social capital in the recovery of severe mental illness: a synthesis of qualitative research. *J. Ment. Heal.* 28 331–339. 10.1080/09638237.2018.1466033 29750586

[B82] Schaefer D. R. Light J. M. Fabes R. A. Hanish L. D. Martin C. L. (2010). Fundamental principles of network formation among preschool children. *Soc. Netw.* 32 61–71. 10.1016/j.socnet.2009.04.003 20161606 PMC2811339

[B83] Schaufeli W. B. Martínez I. M. Pinto A. M. Salanova M. Bakker A. B. (2002). Burnout and Engagement in University Students. *J. Cross. Cult. Psychol.* 33 464–481. 10.1177/0022022102033005003

[B84] Shaw L. H. Gant L. M. (2002). In Defense of the Internet: the Relationship between Internet Communication and Depression, Loneliness, Self-Esteem, and Perceived Social Support. *Cyber Psychol. Behav.* 5 157–171. 10.1089/109493102753770552 12025883

[B85] Shin Y. (2007). Peer Relationships, Social Behaviours, Academic Performance and Loneliness in Korean Primary School Children. *Sch. Psychol. Int.* 28 220–236. 10.1177/0143034307078103

[B86] Simons M. Lataster J. Reijnders J. Peeters S. Janssens M. Jacobs N. (2020). Bonding personal social capital as an ingredient for positive aging and mental well-being. A study among a sample of Dutch elderly. *Aging Ment. Health* 24 2034–2042. 10.1080/13607863.2019.1650887 31389250

[B87] Smirnov I. Thurner S. (2017). Formation of homophily in academic performance: students change their friends rather than performance. *PLoS One* 12:e0183473. 10.1371/journal.pone.0183473 28854202 PMC5576666

[B88] Snijders T. A. B. (2001). The Statistical Evaluation of Social Network Dynamics. *Sociol. Methodol.* 31 361–395. 10.1111/0081-1750.00099

[B89] Steglich C. Snijders T. A. B. Pearson M. (2010). Dynamic Networks And Behavior: separating Selection From Influence. *Sociol. Methodol.* 40 329–393. 10.1111/j.1467-9531.2010.01225.x

[B90] Stokes J. P. (1985). The relation of social network and individual difference variables to loneliness. *J. Pers. Soc. Psychol.* 48 981–990. 10.1037/0022-3514.48.4.981

[B91] Stokes J. P. Levin I. (1986). Gender differences in predicting loneliness from social network characteristics. *J. Pers. Soc. Psychol.* 51 1069–1074. 10.1037/0022-3514.51.5.1069 3794995

[B92] Subrahmanyam K. Greenfield P. (2008). Online Communication and Adolescent Relationships. *Futur. Child.* 18 119–146. 10.1353/foc.0.0006 21338008

[B93] Thomas L. Orme E. Kerrigan F. (2020). Student Loneliness: the Role of Social Media Through Life Transitions. *Comput. Educ.* 146:103754. 10.1016/j.compedu.2019.103754

[B94] Tinto V. (1993). *Leaving College: Rethinking the Causes and Cures of Student Attrition*, 2nd editio Edn. Chicago: University of Chicago Press.

[B95] UNESCO (2020). *UNESCO Rallies International Organizations, Civil Society and Private Sector Partners in a Broad Coalition to Ensure LearningNeverStops.* Available online at: https://en.unesco.org/news/unesco-rallies-international-organizations-civil-society-and-private-sector-partners-broad (accessed September 27, 2021).

[B96] UNESCO (2021). *Global Education Coalition.* Available online at: https://en.unesco.org/covid19/educationresponse/globalcoalition (accessed September 27, 2021).

[B97] Valkenburg P. M. Peter J. (2007a). Online Communication and Adolescent Well-Being: testing the Stimulation Versus the Displacement Hypothesis. *J. Comput. Commun.* 12 1169–1182. 10.1111/j.1083-6101.2007.00368.x

[B98] Valkenburg P. M. Peter J. (2007b). Preadolescents’ and adolescents’ online communication and their closeness to friends. *Dev. Psychol.* 43 267–277. 10.1037/0012-1649.43.2.267 17352538

[B99] Van Baarsen B. Smit J. H. Snijders T. A. B. Knipscheer K. P. M. (1999). Do personal conditions and circumstances surrounding partner loss explain loneliness in newly bereaved older adults? *Ageing Soc.* 19 441–469. 10.1017/S0144686X9900745X

[B100] van Baarsen B. Snijders T. A. B. Smit J. H. van Duijn M. A. J. (2001). Lonely but Not Alone: emotional Isolation and Social Isolation as Two Distinct Dimensions of Loneliness in Older People. *Educ. Psychol. Meas.* 61 119–135. 10.1177/00131640121971103

[B101] Watkins M. W. (2017). The reliability of multidimensional neuropsychological measures: from alpha to omega. *Clin. Neuropsychol.* 31 1113–1126. 10.1080/13854046.2017.1317364 28429633

[B102] Weiss R. S. (1973). *Loneliness: the Experience of Emotional and Social Isolation.* Cambridge: MIT Press.

[B103] Wickham H. (2016). *ggplot2 Elegant Graphics for Data Analysis.* Available online at: https://ggplot2.tidyverse.org (accessed October 18, 2020).

[B104] Yang K. Victor C. (2011). Age and loneliness in 25 European nations. *Ageing Soc.* 31 1368–1388. 10.1017/S0144686X1000139X

[B105] Yarcheski A. Mahon N. E. Yarcheski T. J. (2011). Stress, Hope, and Loneliness in Young Adolescents. *Psychol. Rep.* 108 919–922. 10.2466/02.07.09.PR0.108.3.919-922 21879638

[B106] Zhou W.-X. Sornette D. Hill R. A. Dunbar R. I. M. (2005). Discrete hierarchical organization of social group sizes. *Proc. R. Soc. B Biol. Sci.* 272 439–444. 10.1098/rspb.2004.2970 15734699 PMC1634986

