# Supplementary material for: The Structure of Social Networks and Its Link to Higher Education Students’ Socio-Emotional Loneliness During COVID-19
Source: Front Psychol. 2022 Jan 13;12:733867. doi: 10.3389/fpsyg.2021.733867 (PMC8792991; doi:10.3389/fpsyg.2021.733867)
Supplement: Supplementary file 1 [file Data_Sheet_1.pdf]

Appendix

1 Students' Close Contact Types

To derive the students' close contact types, we asked them several questions about their named close contacts. These responses were used as the basis for typification and subsequent assessment of contact homogeneity. The following variables were queried individually for each contact mentioned.

Contact Type. The question "How did the exchange with this person predominantly take place during the last four weeks?" had the following two response options: 1 = "predominantly online (e.g., via smartphone, tablet, laptop, or PC; also, telephone contact)" and 2 = "predominantly offline (personal contact)."

Initiation of exchange. The question "Who did predominantly establish this exchange?" had the following three response options: 1 = "I predominantly established the contact (e.g., 'I predominantly chatted this person up or suggested a face-to-face meeting')", 2 = "This person predominantly established contact with me (e.g., 'this person has predominantly chatted me up or suggested a face-to-face meeting')" and 3 = "Neither."

Gender. Here, students could select 1 = "male," 2 = "female," and 3 = "other" for their individual contacts.

Residence. For place of residence, students could indicate for each of their contacts whether they lived "in the same household," "in the same district," "in the same town," "in another town," or "in another country."

Relationship to student. To assess the relationship to the contact person, the type of relationship that existed between contact and student was assessed. There were seven possible answers: "parent", "sibling", "relative", "partner", "friend", "fellow student", "other".

Social attraction. To measure social attraction between the students and their contacts, we used an appropriate item with a good factor loading of .70 from the social attraction factor of McCroskey and McCain's (1974) interpersonal attraction scales. The question, slightly adapted from the original, was: "How much do you agree with the following statement? If I could, I would like to have a friendly conversation with this person again in the near future." A six-point Likert scale with the poles 1 = "not at all true" and 6 = "completely true" was offered as possible answers.

Media Skill. The question raised was, "How would you rate this person's competence in using smartphones (and tablets), laptops and PCs?" The students were offered a six-point scale with the poles "layperson" and "professional," with no intermediate names as possible answers.

In Tables A1a and A1b, the descriptive statistics of the mentioned variables of the listwise complete cases can be found, which were used for further analysis.

Table A1a

Descriptives of Students' Contacts (N = 1,758 Complete Cases, Ordinal Variables).

	M	*SD*	Median	Mad	Min	Max	Skew	Kurtosis
Attraction	5.58	0.83	6.00	0.00	1.00	6.00	-2.69	9.14
Media Skill	4.29	1.42	5.00	1.48	1.00	6.00	-0.65	-0.41

Table A1b

Descriptives of Students' Contacts (N = 1,758 Complete Cases, Categorical Variables).

Variable	Category	Frequency	Percent	Cumulative Percent
Contact Type	Online	833	47.4	47.4
	Offline	925	52.6	100.0
Initiation	I	464	26.4	26.4
	This person	315	17.9	44.3
	Neither	979	55.7	100.0
Gender	Male	673	38.3	38.3
	Female	1070	60.9	99.1
	other	15	0.9	100.0
Residence	Same household	469	26.7	26.7
	Same district	128	7.3	34.0
	Same town	309	17.6	51.5
	Another town	796	45.3	96.8
	Another country	56	3.2	100.0
Relationship	Parent	389	22.1	22.1
	Sibling	143	8.1	30.3
	Relative	65	3.7	34.0
	Partner	178	10.1	44.1
	Friend	794	45.2	89.2
	Fellow Student	134	7.6	96.9
	Other	55	3.1	100.0

1.1 t-Distributed Stochastic Neighbor Embedding Analysis of Students' Contacts

To derive the students' close contact types, we used a two-step process. First, we utilized a novel technique for dimensionality reduction called t-Distributed Stochastic Neighbor Embedding (t-SNE; Hinton and Roweis, 2002). We then used the data generated by t-SNE to perform a k-means cluster analysis.

T-Distributed Stochastic Neighbor Embedding is a non-linear technique for dimensionality reduction which is often used in the visualization of high-dimensional datasets (Van Der Maaten and Hinton, 2008). Through its process, t-SNE can detect clusters in data very well (Linderman and Steinerberger, 2019). Therefore, the results of t-SNE are suitable for identifying best clusters using traditional clustering techniques (Dhalmahapatra et al., 2019; Melit Devassy et al., 2020). This relationship was also observed in our data set. With the t-SNE pretreatment, good cluster solutions could be identified more reliably. In preparation for subsequent cluster analysis, a three-dimensional t-SNE analysis was calculated with the following settings: perplexity = 60, theta = 0.2, exaggeration = 4 and 4,000 iterations calculated. Changing the values had little effect on the subsequent cluster analysis and thus, on the results.

2 Clustering of Students' Contacts

To ultimately label clusters, a k-means cluster analysis was performed using t-SNE result. Hopkins's statistic of 0.85 indicated that the dataset showed a clustering tendency (Hopkins and Skellam, 1954). A k-means cluster analysis was performed using 20 iterations (i.e., "iter.max = 20") and 25 random sets (i.e., "nstart = 25"). The number of clusters was calculated using the gap statistic, as this method is considered to be very reliable (Tibshirani et al., 2001). Here, 1,000 Monte Carlo samples were used. The gap statistics indicated 12 clusters, as shown in Figure A1. The quality of the clusters was assessed by silhouette plots and silhouette coefficient, see Figure A2. Their silhouette plots, as well as the average silhouette width of 0.4 indicated acceptable quality (Kaufman and Rousseeuw, 1990). K-means clustering in combination with the used gap statistics identified 12 clusters of sizes $n_1 = 70$, $n_2 = 64$, $n_3 = 83$, $n_4 = 68$, $n_5 = 67$, $n_6 = 78$, $n_7 = 50$, $n_8 = 85$, $n_9 = 48$, $n_{10} = 77$, $n_{11} = 79$, $n_{12} = 100$. Figure A3 illustrates the clustered t-SNE result.

We performed the process of t-SNE and cluster analysis several times to investigate the robustness of the results. Despite minor changes (e.g., thirteen clusters identified instead of twelve), the regression results changed only minimally (no significant changes in hypothesis-related predictor weights), indicating robustness of the results. Thus, each student received twelve new variables with the frequency of the types that occurred. From these twelve numbers, the Shannon entropy, which is a profound measure of diversity (Jost, 2006), was calculated.

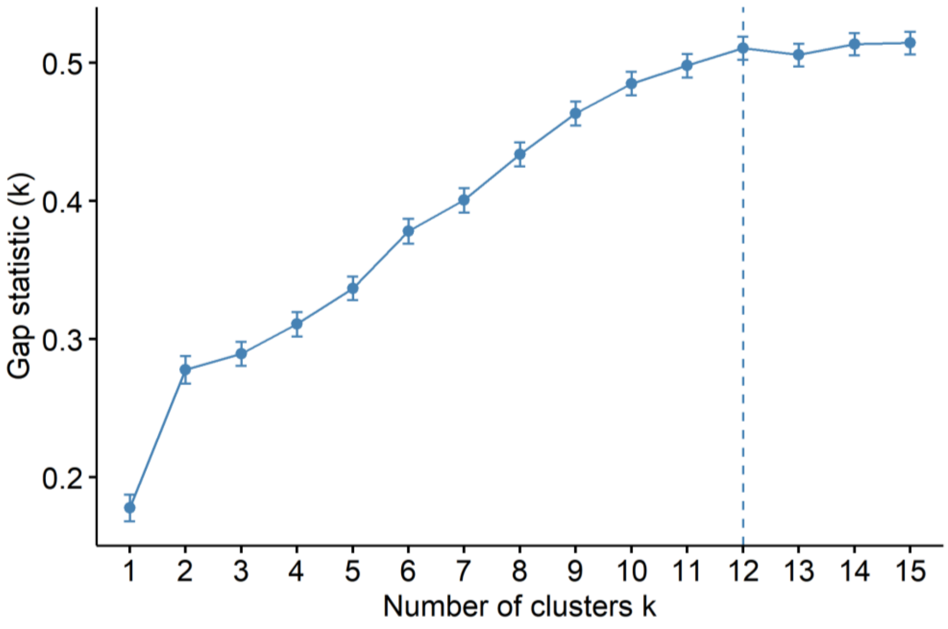

Figure A1. Plot of gap statistics to determine optimal number of clusters.

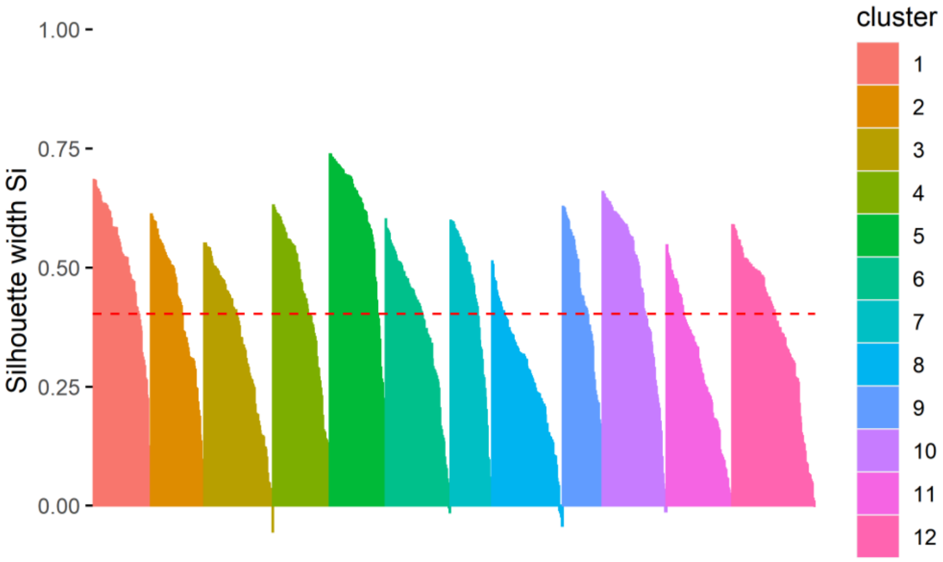

Figure A2. Cluster silhouette plot of twelve clusters with an average silhouette width of .4.

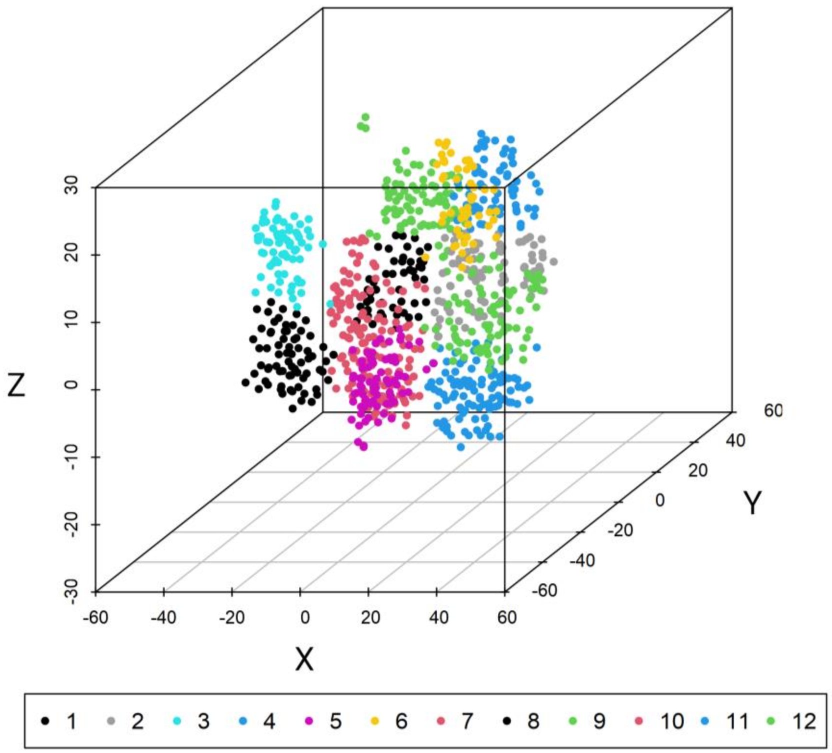

Figure A3. Visualization of the clustered t-SNE result.

3 Complete Regression Tables

Table A2

Regression Results for Social Loneliness as the Criterion.

Predictor	*b*	*b* 95% CI [LL, UL]	β	Fit	Difference
(Intercept)	3.34**	[2.55, 4.12]			
Gender	–0.26*	[–0.53, –0.00]	–.12		
Age	–0.00	[–0.03, 0.02]	–.02		
Offline contacts	–0.12**	[–0.20, –0.04]	–.18		
Partner	–0.10	[–0.35, 0.14]	–.05		
				R^2 = .055** 95% CI[.01,.10]	
(Intercept)	4.62**	[3.54, 5.70]			
Gender	–0.31*	[–0.57, –0.05]	–.14		
Age	–0.00	[–0.03, 0.02]	–.02		
Offline contacts	–0.12**	[–0.20, –0.05]	–.19		
Partner	0.01	[–0.23, 0.26]	.01		
Inf.-sharing beh.	*–0.24***	*[–0.38, –0.10]*	*–.20*		
				R^2 = .092** 95% CI[.03,.15]	ΔR^2 = .037** 95% CI[–.01, .08]
(Intercept)	4.65**	[3.61, 5.69]			
Gender	–0.20	[–0.46, 0.05]	–.09		
Age	0.00	[–0.02, 0.03]	.02		
Offline contacts	–0.19**	[–0.27, –0.12]	–.30		
Partner	–0.04	[–0.28, 0.20]	–.02		
Inf.-sharing beh.	–0.19**	[–0.33, –0.05]	–.16		
Online contacts	*–0.17***	*[–0.24, –0.10]*	*–.29*		
				R^2 = .159** 95% CI[.07,.22]	ΔR^2 = .067** 95% CI[.01, .12]
(Intercept)	5.14**	[4.07, 6.22]			
Gender	–0.14	[–0.39, 0.12]	–.06		
Age	0.00	[–0.02, 0.03]	.02		
Offline contacts	–0.21**	[–0.29, –0.14]	–.33		
Partner	0.05	[–0.20, 0.29]	.02		
Inf.-sharing beh.	–0.20**	[–0.34, –0.06]	–.17		
Online contacts	–0.21**	[–0.28, –0.13]	–.36		
Interconnectedness	*–0.65***	*[–1.07, –0.23]*	*–.18*		
				R^2 = .186** 95% CI[.09,.25]	ΔR^2 = .027** 95% CI[–.01, .06]
(Intercept)	4.96**	[3.89, 6.03]			

gender	–0.12	[–0.37, 0.13]	–.05		
Age	0.00	[–0.02, 0.02]	.01		
Offline contacts	–0.27**	[–0.36, –0.18]	–.41		
Partner	0.04	[–0.20, 0.28]	.02		
Inf.-sharing beh.	–0.18**	[–0.32, –0.05]	–.15		
Online contacts	–0.26**	[–0.34, –0.18]	–.44		
Interconnectedness	–0.80**	[–1.24, –0.37]	–.22		
Heterogeneity	*0.44***	*[0.12, 0.75]*	*.17*		
				R^2 = .208**	ΔR^2 = .021**
				95% CI[.11,.27]	95% CI[–.01, .05]

Note. A significant *b*-weight indicates the beta-weight and semi-partial correlation are also significant. *b* represents unstandardized regression weights. *beta* indicates the standardized regression weights. *LL* and *UL* indicate the lower and upper limits of a confidence interval, respectively. *Inf.-sharing beh.* stands for information-sharing behavior.

* indicates $p < .05$. ** indicates $p < .01$.

Table 5

Regression Results for Emotional Loneliness as the Criterion.

Predictor	*b*	*b* 95% CI [LL, UL]	β	Fit	Difference
(Intercept)	3.83**	[3.05, 4.61]			
Gender	0.01	[–0.26, 0.27]	.00		
Age	–0.03*	[–0.05, –0.00]	–.14		
Offline contacts	–0.03	[–0.11, 0.04]	–.05		
Partner	–0.09	[–0.34, 0.15]	–.05		
				R^2 = .026	
				95% CI[.00,.06]	
(Intercept)	4.52**	[3.43, 5.62]			
Gender	–0.02	[–0.28, 0.25]	–.01		
Age	–0.03*	[–0.05, –0.00]	–.14		
Offline contacts	–0.03	[–0.11, 0.04]	–.05		
Partner	–0.03	[–0.28, 0.22]	–.02		
Inf.-sharing beh.	*–0.13*	*[–0.27, 0.01]*	*–.11*		
				R^2 = .037	ΔR^2 = .011
				95% CI[.00,.07]	95% CI[-.01, .03]
(Intercept)	4.53**	[3.44, 5.62]			
Gender	0.01	[–0.25, 0.28]	.01		
Age	–0.03*	[–0.05, –0.00]	–.13		
Offline contacts	–0.05	[–0.14, 0.03]	–.08		
Partner	–0.05	[–0.30, 0.21]	–.02		
Inf.-sharing beh.	–0.12	[–0.26, 0.03]	–.10		
Online contacts	*–0.05*	*[–0.12, 0.03]*	*–.08*		
				R^2 = .043	ΔR^2 = .006
				95% CI[.00,.08]	95% CI[–.01, .02]
(Intercept)	4.70**	[3.55, 5.85]			
Gender	0.04	[–0.23, 0.31]	.02		
Age	–0.03*	[–0.05, –0.00]	–.12		
Offline contacts	–0.06	[–0.14, 0.02]	–.09		
Partner	–0.02	[–0.28, 0.24]	–.01		
Inf.-sharing beh.	–0.12	[–0.27, 0.03]	–.10		
Online contacts	–0.06	[–0.14, 0.02]	–.11		
Interconnectedness	*–0.23*	*[–0.68, 0.23]*	*–.06*		
				R^2 = .046	ΔR^2 = .003
				95% CI[.00,.08]	95% CI[–.01, .02]
(Intercept)	4.50**	[3.36, 5.64]			
Gender	0.06	[–0.21, 0.33]	.03		
Age	–0.03*	[–0.05, –0.00]	–.13		

Offline contacts	–0.13**	[–0.22, –0.03]	–.19		
Partner	–0.03	[–0.29, 0.23]	–.01		
Inf.-sharing beh.	–0.10	[–0.24, 0.05]	–.08		
Online contacts	–0.12**	[–0.21, –0.03]	–.20		
Interconnectedness	–0.40	[–0.86, 0.07]	–.11		
Heterogeneity	*0.49***	*[0.16, 0.83]*	*.20*		
				R^2 = .074**	ΔR^2 = .028**
				95% CI[.01,.11]	95% CI[–.01, .07]

Note. A significant *b*-weight indicates the beta-weight and semi-partial correlation are also significant. *b* represents unstandardized regression weights. *beta* indicates the standardized regression weights. *LL* and *UL* indicate the lower and upper limits of a confidence interval, respectively. *Inf.-sharing beh.* stands for information-sharing behavior.

* indicates $p < .05$. ** indicates $p < .01$.

4 Appendix References

Dhalmahapatra, K., Shingade, R., Mahajan, H., Verma, A., and Maiti, J. (2019). Decision support system for safety improvement: An approach using multiple correspondence analysis, t-SNE algorithm and K-means clustering. *Comput. Ind. Eng.* 128, 277–289. doi:10.1016/j.cie.2018.12.044.

Hinton, G., and Roweis, S. T. (2002). Stochastic neighbor embedding. *NIPS* 12, 833–840.

Hopkins, B., and Skellam, J. G. (1954). A New Method for determining the Type of Distribution of Plant Individuals. *Ann. Bot.* 18, 213–227. doi:10.1093/oxfordjournals.aob.a083391.

Jost, L. (2006). Entropy and diversity. *Oikos* 113, 363–375. doi:10.1111/j.2006.0030-1299.14714.x.

Kaufman, L., and Rousseeuw, P. J. (1990). *Finding Groups in Data*. , eds. L. Kaufman and P. J. Rousseeuw Hoboken, NJ, USA: John Wiley & Sons, Inc. doi:10.1002/9780470316801.

Linderman, G. C., and Steinerberger, S. (2019). Clustering with t-SNE, Provably. *SIAM J. Math. Data Sci.* 1, 313–332. doi:10.1137/18M1216134.

McCroskey, J. C., and McCain, T. A. (1974). The measurement of interpersonal attraction. *Speech Monogr.* 41, 261–266. doi:10.1080/03637757409375845.

Melit Devassy, B., George, S., and Nussbaum, P. (2020). Unsupervised Clustering of Hyperspectral Paper Data Using t-SNE. *J. Imaging* 6, 29. doi:10.3390/jimaging6050029.

Tibshirani, R., Walther, G., and Hastie, T. (2001). Estimating the number of clusters in a data set via the gap statistic. *J. R. Stat. Soc. Ser. B (Statistical Methodol.* 63, 411–423. doi:10.1111/1467-9868.00293.

Van Der Maaten, L., and Hinton, G. (2008). Visualizing data using t-SNE. *J. Mach. Learn. Res.* 9.
